# Therapeutic modulation of inflammasome pathways

**DOI:** 10.1111/imr.12908

**Published:** 2020-08-07

**Authors:** Dhruv Chauhan, Lieselotte Vande Walle, Mohamed Lamkanfi

**Affiliations:** ^1^ Janssen Immunosciences World Without Disease Accelerator Pharmaceutical Companies of Johnson & Johnson Beerse Belgium; ^2^ Laboratory of Medical Innate Immunity Department of Internal Medicine and Pediatrics Ghent University Ghent Belgium

**Keywords:** disease, GSDMD, IL‐1, immunotherapy, inflammasome, inflammation, NLRP3, pyroptosis

## Abstract

Inflammasomes are macromolecular complexes formed in response to pathogen‐associated molecular patterns (PAMPs) and danger‐associated molecular patterns (DAMPs) that drive maturation of the pro‐inflammatory cytokines interleukin (IL)‐1β and IL‐18, and cleave gasdermin D (GSDMD) for induction of pyroptosis. Inflammasomes are highly important in protecting the host from various microbial pathogens and sterile insults. Inflammasome pathways are strictly regulated at both transcriptional and post‐translational checkpoints. When these checkpoints are not properly imposed, undue inflammasome activation may promote inflammatory, metabolic and oncogenic processes that give rise to autoinflammatory, autoimmune, metabolic and malignant diseases. In addition to clinically approved IL‐1‐targeted biologics, upstream targeting of inflammasome pathways recently gained interest as a novel pharmacological strategy for selectively modulating inflammasome activation in pathological conditions.

## 1 INTRODUCTION

Our innate immune system orchestrates a first and rapid response against microbial pathogens and sterile damage. Innate immune cells respond to these threats by activation of non‐specific germline‐encoded pattern recognition receptors (PRRs).^1^ Some PRRs directly bind evolutionary conserved moieties from pathogens that are commonly referred to as pathogen‐associated molecular patterns (PAMPs) or respond to danger‐associated molecular patterns (DAMPs) that are generated or dislocated upon cellular damage. Other PRRs indirectly guard against infections by detecting the activity of virulence factors used by a suite of pathogens to manipulate the host cell machinery in order to survive in a hostile intracellular environment. Toll‐like receptors (TLRs) and c‐type lectin receptors (CLRs) are examples of membrane‐bound PRRs that survey the extracellular environment and the endosomal compartment. Other PRRs are expressed intracellularly or face the cytosolic compartment, such as the nucleotide‐binding domain, leucine‐rich repeat‐containing proteins (NLRs), the retinoic acid‐inducible gene‐I (RIG‐I)‐like receptors (RLRs), absent in melanoma 2 (AIM2)‐like receptors (ALRs) and members of the tripartite motif (TRIM) receptors.^1^

Most PRRs engage signaling pathways that result in extensive transcriptional reprogramming of the cell, including upregulation of additional PRRs and production of pro‐inflammatory cytokines, chemokines and type I interferons that orchestrate the early inflammatory and host defense responses and help to shape downstream activation of adaptive immunity.^2^ In contrast, a subset of NLRs (NLRP3, NLRC4, and NLRP1), ALRs (AIM2), and Pyrin (a TRIM family member) regulate innate immune responses at the post‐translational level by assembling inflammasome complexes.^3^, ^4^ Although these inflammasome sensors differ in their expression patterns and the upstream signaling pathways that regulate their activation, they all oligomerize and recruit the adapter molecule apoptosis‐associated speck‐like protein containing a CARD (ASC) to seed a fibrillary structure named the “ASC speck” that recruits caspase‐1 through homotypic pyrin‐pyrin (PYD‐PYD) and CARD‐CARD interactions.^3^, ^4^ These inflammasome complexes serve to facilitate proximity‐induced autoactivation of caspase‐1, which proceeds to cleave the pro‐inflammatory cytokines interleukin (IL)‐1β and IL‐18 into bioactive, secreted cytokines. Besides the amino‐terminal CARD domain by which it is recruited into inflammasomes, caspase‐1 consists of a carboxy‐terminal protease domain that harbors the evolutionary conserved catalytic residues in its p20 and p10 subunits.^5^ Interestingly, naturally occurring genetic variants in human caspase‐1 that decrease its protease activity and the levels of IL‐1β production have been associated with the development of autoinflammatory disease in patients.^6^ Recent studies in mice expressing a catalytically inactive caspase‐1‐C284A mutant suggest that inactive caspase‐1 may recruit receptor‐interacting protein kinase 2 (RIPK2) to enhance NF‐κB‐mediated IL‐6 and TNF‐α production.^7^ Additionally, inflammasome activation promotes ASC‐ and caspase‐8‐mediated apoptosis in macrophages expressing catalytically inactive caspase‐1, suggesting that nature evolved mechanisms to safeguard inflammatory responses when caspase‐1 activation is compromised.^8^

Concomitant with cleavage of IL‐1β and IL‐18, caspase‐1 activation results in a lytic regulated cell death mode, termed pyroptosis, through proteolytic activation of gasdermin D (GSDMD).^9^, ^10^, ^11^ Upon cleavage, the pore‐forming amino‐terminal domain of GSDMD inserts into the plasma membrane, where it assembles into higher order oligomers that perforate the plasma membrane and eventually result in osmotic swelling and cell rupture.^12^, ^13^, ^14^ GSDMD‐mediated cell lysis promotes the extracellular release of IL‐1β and IL‐18 from macrophages along with other alarmins such as IL‐1α and high mobility group protein B1 (HMGB1) that contribute to shaping innate immune and inflammatory responses.^9^, ^10^, ^11^, ^15^, ^16^, ^17^, ^18^

In this review, we will briefly introduce the different inflammasome pathways followed by a discussion of IL‐1‐targeted biologics that are currently approved for the treatment of specific autoinflammatory diseases. Finally, we will provide an overview of experimental biologics and small molecule inflammasome agonist and antagonist therapies that are under development for modulating inflammasome activation in inflammatory and malignant diseases.

## 2 INFLAMMASOME SENSORS AND THEIR ACTIVATION MECHANISMS

### 2.1 NLRP1

**FIGURE 1:**
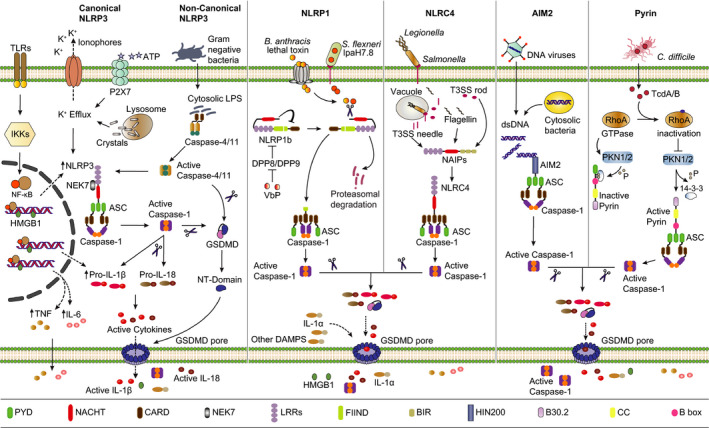
Overview of different inflammasome complexes: (From left to right) Engagement of different TLR receptors leads to activation of IKKs, which in turn results in activation of NF‐κB signaling. Consequently, NF‐κB upregulates different pro‐inflammatory cytokines such as TNF, IL‐6, and pro‐IL‐1β. In addition, NF‐κB signaling upregulates NLRP3 expression (priming step). In the activation step of the NLRP3 inflammasome, diverse stimuli such as nigericin (ionophore), extracellular ATP, and medically relevant crystals (MSU, cholesterol) are sensed by NLRP3 and trigger its activation. Given the diversity of stimuli that activate NLRP3, K^+^ efflux has been proposed as a common mechanism for NLRP3 inflammasome activation, although K^+^ efflux‐independent pathways also exist. NEK7 protein is an essential mediator for NLRP3 activation that acts independently of its kinase activity. NLRP3 recruits the adapter protein ASC and the protease caspase‐1, which drives maturation of pro‐IL‐1β and pro‐IL‐18 into their respective active forms. At the same time, caspase‐1 cleaves GSDMD to release GSDMD‐N that forms GSDMD pores into the plasma membrane and drives pyroptosis, causing the release of various DAMPs such as IL‐1β, IL‐18, IL‐1α, and HMGB1. In the non‐canonical NLRP3 pathway, intracellular sensing of LPS activates caspase‐4/11 (human/mouse) that cleaves GSDMD, which induces pyroptosis, consequently activating the NLRP3 inflammasome and IL‐1β maturation through K^+^‐efflux. NLRP1 undergoes auto‐catalytic processing in its FIIND domain. Proteasomal degradation of its auto‐inhibitory N‐terminus is considered a primary requirement of NLRP1 inflammasome activation. In rodents, cleavage on the N‐terminal part of the NLRP1b protein by anthrax lethal toxin or IpaH7.8 allows the C‐terminal CARD domain to engage caspase‐1 for activation. In addition, mouse NLRP1b and human NLRP1 are activated by DPP8/DPP9 inhibitors. Activation of the NLRC4 inflammasome requires NAIPs that recognize T3SS inner rod proteins, needle proteins, or flagellin. In case of NLRP1b and NLRC4, caspase‐1 can directly be recruited independently of the adapter ASC. dsDNA of microbial or host origin in the cytosolic compartment activates AIM2, which requires ASC to activate caspase‐1. *Clostridium difficile* toxins (TcdA/B) inhibit RhoA GTPases activity, which inhibits PKN1/2‐dependent phosphorylation of Pyrin. Consequently, 14‐3‐3 disengages from Pyrin, allowing the assembly of the Pyrin inflammasome

The first member of the NLR family shown to form an inflammasome is NLRP1.^19^ Humans encode a single *NLRP1* gene, whereas mice carry three paralogous genes named *Nlrp1a*, *Nlrp1b*, and *Nlrp1c*. The latter is considered a pseudogene, whereas both *NLRP1a* and *NLRP1b* assemble inflammasomes.^20^, ^21^ *Bacillus anthracis* lethal toxin (LeTx) specifically activates the NLRP1b inflammasome, while agents that selectively engage the *NLRP1a* inflammasome remain to be discovered. The cytosolic postproline dipeptidyl peptidases (DPP)8 and DPP9 suppress activation of human NLRP1 and its murine orthologs through mechanisms that are incompletely understood. In agreement, boronic acid peptides such as Val‐boroPro (also known as Talabostat) that inhibit DPP8/9 as well as several other DPP family members, and the DDP8/9‐selective inhibitor 1G244 activate human NLRP1 and the murine NLRP1b inflammasomes.^22^, ^23^, ^24^, ^25^ Activation of human NLRP1 and murine NLRP1b is thought to involve proteasomal degradation of the auto‐inhibitory amino‐terminal region, which enables the recruitment of ASC and caspase‐1 to their carboxy‐terminal UPA‐CARD‐containing regions (Figure 1).^26^, ^27^

A homozygous NLRP1 gain‐of‐function mutation was recently reported to cause an autosomal recessive form of juvenile‐onset recurrent respiratory papillomatosis in two siblings with mild dermatologic abnormalities.^28^ However, dominantly inherited gain‐of‐function mutations in human *NLRP1* predispose patients to the development of Mendelian diseases characterized by skin inflammation and dyskeratosis named multiple self‐healing palmoplantar carcinoma (MSPC), *NLRP1*‐associated autoinflammation with arthritis and dyskeratosis (NAIAD), and autoinflammation with arthritis and dyskeratosis (AIADK).^29^, ^30^

### 2.2 NLRP3

Due to its broad role in anti‐microbial immunity and sterile inflammation, NLRP3 is subjected to multilayered regulation at the transcriptional, post‐translational, and activation levels. The protein has a tripartite domain architecture composed of an amino‐terminal PYD, a central NACHT domain, and a C‐terminal region containing leucine‐rich repeat (LRR) motifs. The NACHT domain features an (d)ATP binding pocket with extended Walker A/B motifs that supports ATP hydrolysis, a prerequisite for activation of the NLRP3 inflammasome.^31^ In naive macrophages, NLRP3 expression levels are insufficient to support inflammasome activation, and NLRP3 activation therefore first requires a transcriptional priming signal (signal 1) to increase NLRP3 protein expression levels (Figure 1).^32^ Priming may additionally involve post‐translational modifications such as phosphorylation and deubiquitination to license rapid subsequent NLRP3 activation by signal 2 agents.^33^, ^34^, ^35^, ^36^, ^37^ Moreover, both the chaperone Hsp90 and the cell cycle‐regulated kinase NIMA‐related kinase 7 (NEK7) physically interact with and regulate NLRP3 activation, although TAK1‐dependent post‐translational priming may also license NEK7‐independent NLRP3 activation in human macrophages.^38^, ^39^, ^40^, ^41^, ^42^

Signal 2 agents are chemically and structurally diverse, ranging from extracellular ATP, microbial pore‐forming protein toxins, and organic ionophores to fine particulate matter such as silica and uric acid crystals. It is therefore thought that NLRP3 senses these agents indirectly through their effect on disruption of the plasma membrane, and the concomitant efflux of intracellular potassium levels.^43^, ^44^, ^45^ NLRP3‐activating agents such as crystalline monosodium urate (MSU), calcium pyrophosphate dehydrate and cholesterol, and protein aggregates like amyloid‐β and islet amyloid polypeptide (IAPP) are implicated in the development of inflammatory pathology in patients. Correspondingly, a rich body of preclinical studies showed that *Nlrp3* deficiency in mice protects against inflammatory pathology in models of gout, pseudogout, atherosclerosis, inflammatory arthritis, non‐alcoholic steatohepatitis (NASH), Alzheimer's disease (AD), and the experimental autoimmune encephalomyelitis (EAE) model of multiple sclerosis.^46^, ^47^, ^48^, ^49^, ^50^, ^51^, ^52^, ^53^, ^54^ Of note, NLRP3 inflammasome activation by influenza A virus (IAV) infection is regulated by the Z‐DNA binding protein 1 (ZBP1)‐containing PANoptosome, a recently identified multi‐protein complex that senses IAV and controls induction of pyroptosis, apoptosis, and necroptosis in IAV‐infected cells.^55^ TAK inhibition and a host of additional microbial pathogens also induce NLRP3 inflammasome‐mediated pyroptosis concomitant with induction of necroptosis and extrinsic apoptosis in the targeted cell population, a process that was recently coined as PANoptosis.^56^, ^57^, ^58^, ^59^

Moreover, gain‐of‐function mutations in NLRP3 cause autosomal dominantly inherited autoinflammatory diseases that are collectively named Cryopyrin‐Associated Periodic Syndrome (CAPS), and which comprise—in increasing order of clinical severity—familial cold‐induced autoinflammatory syndrome (FCAS), Muckle‐Wells syndrome (MWS), and neonatal‐onset multisystem inflammatory disease (NOMID) (also known as chronic infantile neurologic, cutaneous, articular syndrome (CINCA)). Mechanistic studies in CAPS mouse models and clinical experience with IL‐1‐targeted therapies both suggest that excessive IL‐1β production is the key mediator of systemic inflammatory pathology in CAPS, with evidence for additional roles of IL‐18 and pyroptosis.^18^, ^60^, ^61^

### 2.3 NLRC4

The NLRC4 inflammasome assembles upon physical binding of bacterial flagellin and type III secretion systems (T3SS) components by NLR family apoptosis inhibitory proteins (NAIPs). Human NAIP recognizes the T3SS needle protein, whereas murine NAIP5 and NAIP6 detect flagellin, and NAIP1 and NAIP2 detect needle and rod proteins of T3SS, respectively (Figure 1).^62^, ^63^, ^64^ Gain‐of‐function mutations in *NLRC4* cause potentially lethal periodic fever syndromes that are characterized by high circulating IL‐18 levels and increased risk for the development of macrophage activation syndrome (MAS).^65^, ^66^

### 2.4 AIM2

The ALR family member *AIM* *2* mediates inflammasome responses upon recognition of double‐stranded DNA (dsDNA) in the cytosol of murine macrophages. It does not appear to select for specific nucleic acid sequences and the detected dsDNA fragments may originate from the host's nuclear genome, or may originate from infection with specific viruses (vaccinia virus and cytomegalovirus) or bacteria (*Francisella tularensis* and *Listeria monocytogenes*) (Figure 1).^67^, ^68^, ^69^, ^70^, ^71^ GTPases of the guanylate‐binding proteins (GBP) family mediate activation of the AIM2 inflammasome by *F. tularensis* and other bacterial pathogens.^72^, ^73^ The *AIM2* inflammasome has also been shown to promote irradiation‐induced hematopoietic failure and gastrointestinal syndrome in response to genomic dsDNA breaks in mice.^74^ Notably, the NLRP3 inflammasome promotes secretion of IL‐1β in human primary monocytes following recognition of cytosolic dsDNA by the cGAS‐STING pathway.^75^ This may explain why contrary to other inflammasome sensors, gain‐of‐function mutations in *AIM2* have not been reported to cause monogenic autoinflammatory disorders in patients.

### 2.5 Pyrin

The Pyrin inflammasome indirectly senses inhibition of RhoA GTPase activity by *Clostridium difficile* toxin A (TcdA) and toxin B (TcdB) and other bacterial toxins.^76^, ^77^, ^78^ Pyrin phosphorylation (at S208/S242 in human Pyrin; S205/S241 in murine Pyrin) keeps the protein in an inactive state in naïve macrophages.^77^, ^79^ RhoA GTPase inhibition results in dephosphorylation of Pyrin, releasing it from 14‐3‐3 proteins and allowing ASC recruitment and subsequent caspase‐1 activation (Figure 1). In addition, microtubules are required for inflammasome activation downstream of Pyrin dephosphorylation.^77^, ^78^ Gain‐of‐function mutations in the *MEFV* gene that encodes Pyrin cause Familial Mediterranean Fever (FMF), a monogenic autoinflammatory disease that affects an estimated 150,000 patients worldwide.^60^ FMF patients present with recurrent fevers associated with serositis and may develop serum amyloid A (SAA) amyloidosis if colchicine therapy is not initiated in time. Notably, disease‐penetrant FMF mutations were recently shown to render Pyrin inflammasome activation independent of microtubules.^78^, ^80^

### 2.6 The non‐canonical inflammasome

Whereas caspase‐1 is the central effector protease in canonical inflammasome pathways, caspases 4 or 5 in humans, and the orthologous caspase‐11 in rodents, exert this role in the non‐canonical inflammasome pathway that is engaged upon cytosolic detection of lipopolysaccharides (LPS)—a major component of the outer membrane of Gram‐negative bacteria.^81^, ^82^, ^83^, ^84^, ^85^, ^86^ These inflammatory caspases cleave GSDMD to induce pyroptosis, and the resulting potassium efflux is thought to result in secondary activation of the NLRP3 inflammasome (Figure 1).^10^, ^81^, ^85^, ^86^ Inhibition of the non‐canonical inflammasome pathway has gained considerable traction as a potential therapeutic strategy in Gram‐negative infections and sepsis ^9^, ^12^, ^82^, ^86^, ^87^, ^88^, ^89^

## 3 IL‐1‐TARGETED THERAPIES IN THE CLINIC

Therapeutic targeting of IL‐1 family cytokines, and IL‐1β in particular, has gained significant traction due to its central role in a host of autoinflammatory diseases.^90^, ^91^ Moreover, results from both clinical trials and preclinical studies implicate IL‐1β in a suite of metabolic, malignant, and neurodegenerative diseases.^90^, ^91^, ^92^ IL‐1β is a highly inflammatory and pyrogenic cytokine, and its production and activity are tightly regulated. Cells of the myeloid lineage—the primary source of IL‐1β in the body—do not express IL‐1β in the absence of inflammatory cues, and first need to transcriptionally upregulate its production in the context of infection or tissue damage. Rather than being secreted through the conventional secretory pathways that govern secretion of most other cytokines, activated myeloid cells produce IL‐1β as a biologically inert precursor (proIL‐1β) that is stored in the cytosol.^93^ It gains biological activity following caspase‐1‐mediated removal of its amino‐terminal pro‐peptide. Once released into the extracellular space, mature IL‐1β binds to IL‐1 receptor 1 (IL‐1R1) on effector cells, which promotes heterodimerization of the receptor with IL‐1R accessory protein (IL‐1RAcP) and the subsequent recruitment of intracellular signaling components such as myeloid differentiation primary response gene 88 (MyD88). This process initiates a signaling cascade ultimately resulting in the activation of NF‐κB and mitogen‐activated protein kinase (MAPK) signaling (Figure 2). Because of its extensive functions and potency, IL‐1 activity in the extracellular environment is further regulated by two mechanisms. Firstly, the IL‐1R antagonist (IL‐1Ra) binds to IL‐1R1 to block binding of IL‐1α and IL‐1β to IL‐1R1. Binding of IL‐1Ra to IL‐1R1 hampers the heterodimerization with IL‐1RAcP, which prevents initiation of intracellular signaling.^94^ Secondly, the decoy receptor IL‐1R type II (IL‐1R2) that lacks the cytoplasmic TIR domain also binds to IL‐1α and IL‐1β, thereby preventing IL‐1 signaling.^95^

**IGURE 2:**
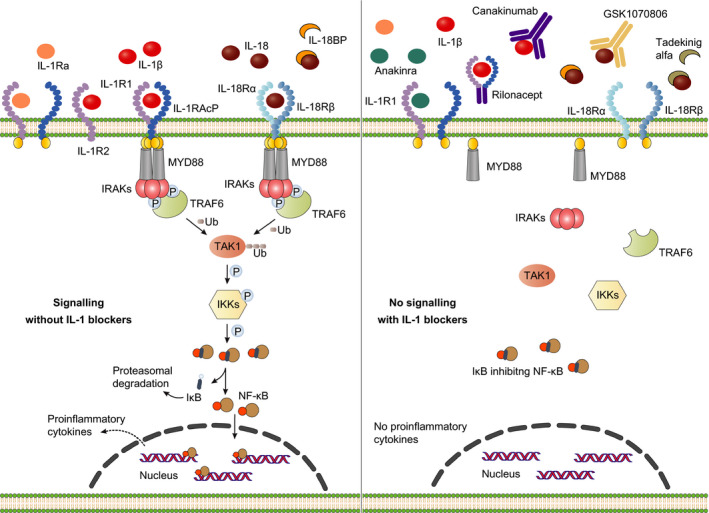
F IL‐1 and IL‐18 signaling and its modulation by therapeutic agents: (Left) Engagement of IL‐1 and IL‐18 receptors by IL‐1α, IL1β, and IL‐18, respectively, leads to the cytosolic recruitment of the TIR domain‐containing adapter MyD88. Subsequently, MyD88 binds to the IRAK kinases IRAK1/2/4, which associate with the E3‐ubiquitin ligase TRAF6. This allows dissociation of IRAK kinases and TRAF6 from the receptor complex and results in the polyubiquitination of the kinase TAK‐1, which leads to the phosphorylation of IKKs. In turn, IKKs phosphorylate and degrade IκB, subsequently inducing the upregulation of NF‐κB target genes and the release of pro‐inflammatory cytokines. To maintain the balance of IL‐1 signaling, extracellular IL‐1Ra competitively inhibits the binding of active IL‐1β and IL‐1α to IL‐1R1. Furthermore, the decoy receptor IL‐1R2 binds to IL‐1β and IL‐1α and inhibits IL‐1 signaling. Similarly, IL‐18 signaling is regulated by the inhibitory activity of IL‐18BP that binds IL‐18 and prevents its interaction with its cell surface receptors. (Right) Three biologic agents targeting IL‐1 signaling have been approved by the FDA. As a recombinant form of the IL‐1Ra, anakinra competes with IL‐1β and IL‐1α for binding to IL‐1R1. The human anti‐IL‐1β monoclonal antibody canakinumab specifically binds and neutralizes IL‐1β to prevent its interaction with IL‐1R1. Rilonacept is a soluble decoy receptor, which consists of the extracellular domains of IL‐1R1 and IL‐1RAcP, and binds and neutralizes both IL‐1β and IL‐1α. Similar to IL‐1 blocking agents, the recombinant IL‐18BP, tadekinig alfa, and the human anti‐IL‐18 monoclonal antibody, GSK1070806, bind to IL‐18 and inhibit the interaction of IL‐18 to its cell surface receptors. Both IL‐18 blockers are currently under investigation in clinical trials

Dependent on the cell type, IL‐1 signaling will result in the production of pro‐inflammatory cytokines, such as TNF‐α, IL‐8, and IL‐6; and chemokines that stimulate the attraction of macrophages and other immune cells, such as monocyte chemoattractant protein 1 (MCP1) and the neutrophil attractant chemokines CXCL1 and MIP‐2. Moreover, IL‐1β is a potent driver of IL‐6‐dependent production of acute phase proteins in hepatocytes, of which C‐reactive protein (CRP) and SAA are two major representatives.^96^ In addition to its central role in innate immune and inflammatory responses, IL‐1β drives polarization of CD4^+^ T cells toward T‐helper type (Th) 1 and Th17 cells, and induces expansion and differentiation of antigen‐specific CD8^+^ T cells.^97^, ^98^ IL‐1β has been attributed a largely beneficial role in resolving acute inflammation. However, several autoinflammatory syndromes are driven by excessive or chronically dysregulated IL‐1 signaling, and IL‐1‐targeted biologics have proven highly effective in treating inflammasomopathies.^60^ Three biologic anti‐IL‐1 agents are currently approved for clinical use: anakinra, canakinumab, and rilonacept (Figure 2).

Anakinra is a nonglycosylated recombinant version of human IL‐1Ra, and functions as a competitive inhibitor of both IL‐1α and IL‐1β for binding to IL‐1R1. It has a good safety profile, but its short half‐life of 4‐6hours makes daily subcutaneous injections necessary to maintain high target engagement levels. Anakinra was initially approved for the treatment of rheumatoid arthritis with methotrexate in patients that failed treatment on methotrexate alone, but these patients are nowadays prioritized to TNF‐blocking agents. Anakinra is nowadays primarily used to treat CAPS and colchicine‐resistant FMF.^60^ Recent clinical studies also support its efficacy in gout showing that the drug is equally effective in relieving pain associated with acute gout flares compared to the standard care of treatment.^99^ A consensus guideline issued in 2012 also recommended anakinra as a treatment for Schnitzler's syndrome, an autoinflammatory disorder with CAPS‐like symptoms that include urticarial, periodic fever and joint and bone pain. Moreover, in a small multicentre, open‐label, randomized clinical trial that compared anakinra with a host of TNF‐α blockers, IL‐1 inhibition with anakinra also proved effective in improving glycemic and inflammatory parameters in patients affected with rheumatoid arthritis and type 2 diabetes.^100^

Canakinumab is a human monoclonal antibody with an extended half‐life of 26 days that specifically binds and neutralizes IL‐1β. It has been approved by the FDA for the treatment of CAPS. Canakinumab is also being used for treating autoinflammatory diseases such as FMF, TNF receptor‐associated periodic syndrome (TRAPS) and hyperimmunoglobulin D syndrome (HIDS)/mevalonate kinase deficiency (MKD), systemic juvenile idiopathic arthritis, and adult onset Still's disease.^101^ More recently, canakinumab was evaluated in the Canakinumab Anti‐inflammatory Thrombosis Outcome Study (CANTOS) (ClinicalTrials.gov Identifier: NCT01327846). This multi‐center, randomized, double‐blind phase 3 trial involved more than 10,000 atherosclerosis patients with previous myocardial infarction and a persistent pro‐inflammatory response, defined by circulating levels of the acute phase protein CRP of 2mg/L or higher.^102^ High sensitivity CRP measurements are approved as a clinical biomarker of cardiovascular risk, and CANTOS was designed to evaluate the therapeutic potential of canakinumab in reducing the risk of cardiovascular events and secondary stroke in this cohort.^103^ Notably, results from the study showed that IL‐1β inhibition with canakinumab reduced serum levels of CRP in a subset of patients. Moreover, a 25% reduction in major adverse cardiovascular events (MACE) and 31% reduction in cardiovascular mortality and all‐cause mortality was observed in patients who achieved CRP levels lower than 2 mg/L, whereas no significant benefit was observed in patients that retained CRP levels of 2 mg/L or above on canakinumab.^103^ In a separate analysis of the CANTOS cohort, the investigators detected a remarkable dose‐dependent reduction in the incidence of lung cancer and lung cancer‐associated mortality with a relative risk reduction of 67% for lung cancer (HR 0.33 [95% CI: 0.18‐0.59]) and 77% for lung cancer mortality (HR 0.23 [95% CI: 0.10‐0.54]) in patients receiving a 300 mg dose of canakinumab every three months.^104^ These exploratory results suggest that IL‐1β neutralization may contribute importantly to intercepting the health trajectory of lung cancer patients and warrant further confirmation in independent studies.

A third clinically approved IL‐1‐targeted therapy for the treatment of CAPS is rilonacept or IL‐1 Trap.^105^ Rilonacept is a human dimeric fusion protein consisting of the extracellular domains of IL‐1R1 and IL‐1RAcP fused to the Fc fragment of IgG. It functions as a soluble decoy receptor that prevents binding of both IL‐1α and IL‐1β to the IL‐1R1 (Figure 2). This chimeric protein has a half‐life of approximately 1week, bridging those of anakinra and canakinumab. Rilonacept also proved effective in controlling symptoms in a cohort of patients suffering from Schnitzler's syndrome (ClinicalTrials.gov Identifier: NCT01045772).^106^

## 4 CLINICAL EVALUATION OF EXPERIMENTAL IL‐18‐TARGETED THERAPIES

Although proIL‐18 is constitutively expressed in myeloid cells, production of bioactive IL‐18—akin to IL‐1β—requires caspase‐1‐mediated cleavage following inflammasome activation. Once secreted into the extracellular environment, binding of IL‐18 to IL‐18 receptor α (IL‐18Rα) and IL‐18 receptor β (IL‐18Rβ) results in downstream activation of inflammatory signaling in effector cells. Given its pleiotropic immune roles, several mechanisms exist that tightly regulate IL‐18 activity in the extracellular milieu.^60^, ^94^ One such regulatory mechanism that is being exploited for diagnosis and therapy of patients is the neutralization of IL‐18 by the natural IL‐18 binding protein (IL‐18BP), which precludes binding of IL‐18 to its cognate receptors (Figure 2).

Given that the expression ratio of IL‐18 and IL‐18BP is often disturbed in inflammatory diseases, circulating levels of IL‐18BP‐unbound IL‐18 (free IL‐18) have been proposed as a biomarker for disease activity in adult onset Still's disease and systemic‐onset juvenile idiopathic arthritis.^107^, ^108^ Moreover, a recombinant version of human IL‐18BP named “tadekinig alfa” demonstrated a favorable safety profile with early signs of efficacy in a recent phase II clinical trial of patients with adult onset Still's disease (ClinicalTrials.gov Identifier: NCT02398435).^109^ Based on promising data showing successful treatment of life‐threatening inflammation in an NLRC4‐MAS patient ^110^, the therapeutic potential of anti‐IL‐18 therapy with tadekinig alfa is currently being investigated in a single‐arm, open‐label phase 3 trial in patients afflicted with NLRC4‐MAS or autoinflammation due to monogenic XIAP mutations (ClinicalTrials.gov Identifier: NCT03113760) given that both syndromes feature high circulating IL‐18 levels.

GSK1070806 is another biologic that was developed to neutralize IL‐18‐mediated inflammation. Unlike tadekinig alfa, GSK1070806 is a recombinant human IL‐18‐neutralizing antibody with an extended half‐life in circulation. It is currently being tested in a phase 2 clinical study for the treatment of patients with moderate to severe Crohn's disease (ClinicalTrials.gov Identifier: NCT03681067) (Figure 2). If proven effective, these clinical studies may lay the foundation to expand the use of IL‐18‐inhibiting biologics to additional autoinflammatory and autoimmune diseases and expand our understanding of how IL‐18 promotes destructive and potentially life‐threatening inflammation in patients.

## 5 GOING UPSTREAM: POTENTIAL BENEFITS OF INHIBITING INFLAMMASOME COMPONENTS

IL‐1‐ and IL‐18‐targeted biologics have dramatically improved therapeutic outcomes for patients suffering from a subset of autoinflammatory diseases, but these protein‐based products require patients to subject themselves to frequent subcutaneous injections. More importantly, chronic suppression of a key arm of the innate immune system inevitably comes with an increased risk for serious infections. Clinical experience with canakinumab and anakinra provides comforting real‐world evidence that the risk of *Mycobacterium tuberculosis* reactivation and tuberculosis development is significantly lower with IL‐1‐targeted biologics compared to TNF‐α inhibitors, but it is evident that chronic IL‐1 blockade does increase risk of opportunistic infections of the upper airways along with some evidence of increased urinary tract infections with *Escherichia coli* and *Streptococci*.^102^, ^111^ Additional adverse events that are associated with chronic IL‐1 inhibition include neutropenia, low platelet counts, headaches, abdominal pain, diarrhea, urticarial lesions, and inflammation at the site of injection.^100^, ^101^, ^102^

Therapies acting upstream of IL‐1β secretion might show an improved safety and efficacy profile because selective inflammasome inhibition would still allow IL‐1‐driven host defense through non‐targeted inflammasomes, for example, when the patient faces an acute infection, while preventing chronic inflammatory pathology that originate from the silenced inflammasome. At the same time, selective inflammasome targeting may increase therapeutic potency through the simultaneous blockade of IL‐1β, IL‐18 and by preventing pyroptosis‐mediated release of DAMPs such as IL‐1α, HMGB1, and ATP. This notion is supported by empirical evidence from a host of mouse disease models that point to GSDMD‐driven pyroptosis as the key driver of inflammasome‐mediated pathology.^17^, ^18^, ^21^, ^61^, ^112^, ^113^ Finally, small molecule inflammasome inhibitors that can be dosed orally would relief patients from the need of lifelong injections. Hence, a suite of tool compounds and natural compounds have been reported to inhibit inflammasome activation in recent years, and some molecules are currently being evaluated in early clinical studies.

## 6 THE QUEST FOR POTENT AND SELECTIVE NLRP3 INHIBITORS

A vast body of studies has implicated the NLRP3 inflammasome in a host of inflammatory, metabolic, and neurodegenerative diseases, rendering this pathway an attractive target for pharmacological modulation across therapeutic areas.^60^, ^91^, ^92^, ^114^ The first demonstration that selective pharmacological inhibition of the NLRP3 inflammasome is feasible arrived in 2009 with the demonstration that the sulfonylurea‐containing compound glyburide inhibited NLRP3‐induced pyroptosis and IL‐1β secretion without affecting activation of the NLRC4 and NLRP1b inflammasomes.^115^ The half‐inhibitory concentration (IC_50_) of glyburide is in the low μM range, whereas the related diarylsulfonylurea compound MCC950/CRID3 inhibits NLRP3 activation with nM potency.^116^ Like glyburide, MCC950/CRID3 exerts high target selectivity as it inhibits Nlrp3 activation without interfering with the AIM2, NLRC4, Pyrin, and NLRP1b inflammasomes, or altering TLR4‐induced cytokine secretion.^78^, ^116^ More recently, MCC950/CRID3 and related tool compounds were shown to directly target the NACHT domain of NLRP3 in the vicinity of its ATP/dATP binding pocket, and they represent the most potent tool compounds that are currently available for selective NLRP3 inflammasome inhibition in human cells and preclinical rodent studies.^117^, ^118^, ^119^ Consequently, MCC950/CRID3‐mediated inhibition of NLRP3 has shown beneficial effects in mice and other preclinical species that have been subjected to disease models, among which models of atherosclerosis, myocardial infarction, colitis, and skin and airway inflammation.^47^, ^116^, ^120^, ^121^, ^122^, ^123^ Interestingly, MCC950/CRID3 and related diarylsulfonylurea compounds may be substantially less effective in inhibiting CAPS‐related NLRP3 mutants because in vivo MCC950/CRID3 concentrations that potently inhibited NLRP3‐driven inflammation in wildtype mice were shown to be ineffective in two in vivo mouse models of CAPS disease.^119^ Concordantly, MCC950/CRID3‐derived photoaffinity probes labeled the NACHT domain of wildtype NLRP3, but not CAPS‐associated NLRP3 mutants.^119^ These results raise the possibility that MCC950/CRID3‐based therapies may need to be dosed at higher concentrations in CAPS patients to effectively treat inflammatory pathology than is needed to curb inflammatory pathology driven by wildtype NLRP3.

In addition to inflammatory disease, MCC950/CRID3 also improved clinical and molecular disease markers in models of neurodegenerative disease such as Parkinson's disease, Alzheimer's disease, and the EAE model of multiple sclerosis.^52^, ^92^, ^114^, ^124^ While EAE is associated with breakdown of the blood‐brain barrier and enhanced vascular permeability,^125^ it is currently unclear whether MCC950/CRID3 also crosses the blood‐brain barrier in Alzheimer's and Parkinson's disease models to reach clinically relevant concentrations in the central nervous system, or whether it dampens neuroinflammatory pathology indirectly in these models by modulating NLRP3 activity in the periphery.

**FIGURE 3:**
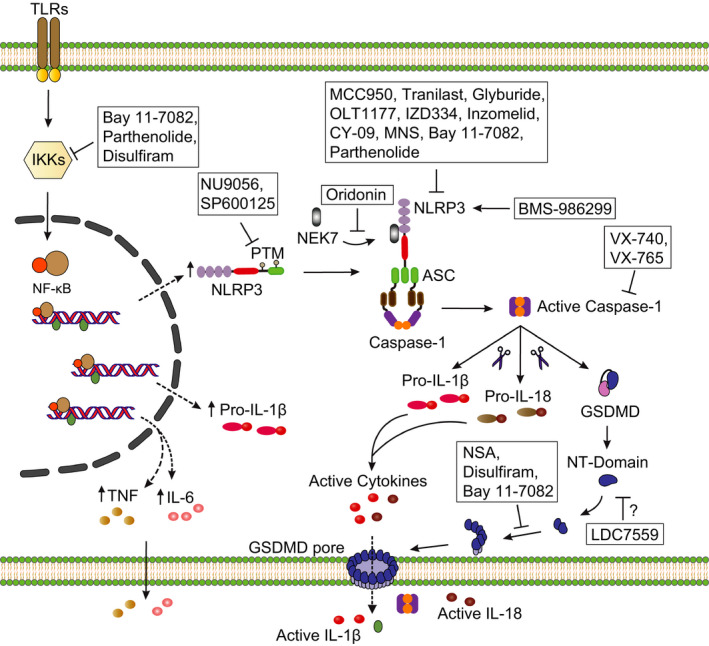
Pharmacological targeting of inflammasomes. NLRP3 inflammasome activation requires a two‐step mechanism. The priming step involves NF‐κB‐dependent transcriptional upregulation of NLRP3, which is activated by the engagement of TLRs, CLRs, and cytokine receptors. Small molecule compounds such as Bay 11‐7082, parthenolide, and disulfiram were shown to inhibit inflammasome signaling at several levels, including the priming step. These compounds inhibit NF‐κB signaling, consequently blocking the expression of pro‐inflammatory cytokines such as pro‐IL‐1β, TNF, and IL‐6, as well as the synthesis of new NLRP3 protein. Small molecules were also shown to inhibit NLRP3 activation at the post‐translational level. The HAT inhibitor, NU9056, was reported to block NLRP3 acetylation, and the JNK kinase inhibitor, SP600125, was shown to inhibit NLRP3 phosphorylation. Other small molecules directly inhibit NLRP3 inflammasome activation. MCC950/CRID3, which is structurally related to the sulfonylurea compound glyburide, is currently the most potent and selective inhibitor of NLRP3 that directly targets its NACHT domain. The MCC950/CRID3‐related compounds IZD334 and Inzomelid are currently under investigation for the treatment of CAPS. CY‐09, MNS, OLT1177, Bay 11‐7082, and parthenolide were shown to bind directly to NLRP3 and inhibit its ATPase activity. Tranilast was shown to inhibit NLRP3 oligomerization via an ATPase‐independent manner. The natural product oridonin prevents the direct interaction between NLRP3 and NEK7. In addition to various inhibitors, the NLRP3 activator, BMS‐986299, is currently being investigated in a clinical trial for cancer treatment. VX‐740 and VX‐765 are specific inhibitors of caspase‐1. Blocking inflammasome signaling by targeting GSDMD is an alternative strategy currently being explored with small molecule inhibitors, such as LDC7559, NSA, Disulfiram, and Bay 11‐7082

Based on these promising results, several MCC950/CRID3 analogs are currently being developed for clinical applications. Two such NLRP3 antagonists, namely IZD334 (ClinicalTrials.gov Identifier: NCT04086602) and Inzomelid (ClinicalTrials.gov Identifier: NCT04015076), have recently completed early clinical testing to assess the safety, tolerability, and pharmacological properties of the drugs in healthy adult participants and to obtain first evidence of clinical efficacy in adult CAPS patients (Figure 3). As a secondary outcome, these studies analyzed NLRP3 activity in stimulated whole blood and monitored clinical symptoms in adult CAPS patients.

Several other small molecule NLRP3 inhibitors have been reported, although the potency and selectivity profile of most are less stringent than that of MCC950/CRID3. For instance, Bay 11‐7082 and parthenolide are anti‐inflammatory compounds that inhibit ATPase activity of NLRP3 and also interfere with NF‐κB‐mediated cytokine production.^126^ Moreover, parthenolide was shown to be a direct inhibitor of recombinant caspase‐1 in vitro^126^; and Bay 11‐7082 and parthenolide were recently also reported to bind and inhibit GSDMD.^127^ These molecules therefore appear less suitable for clinical development as NLRP3‐targeted therapies given the risks associated with broad immunosuppression.

The tryptophan derivative tranilast also inhibits NLRP3 by direct binding to its NACHT domain (Figure 3).^128^ This molecule demonstrated therapeutic efficacy in mouse models of NLRP3‐driven gouty arthritis and CAPS. In addition, tranilast blunted spontaneous caspase‐1 activation and IL‐1β release from isolated synovial fluid mononuclear cells of arthritis patients.^128^ Since its identification in 1982 as an anti‐allergic agent that suppresses histamine release from mast cells, tranilast has been associated with a growing list of anti‐inflammatory effects, including suppression of TGF‐β, NF‐κB, and MAPK signaling (reviewed in 129), suggesting that it may not solely inhibit inflammation by suppressing NLRP3 activation. However, considering its clinical safety, tolerability, and efficacy profile in different indications, tranilast is currently being evaluated in a phase 2 open‐label clinical trial for efficacy and safety in CAPS patients (ClinicalTrials.gov Identifier: NCT03923140).

The β‐sulfonyl nitrile compound OLT1177 also was shown to inhibit NLRP3‐mediated IL‐1β release from stimulated PBMCs of CAPS patients and primary human neutrophils (Figure 3).^130^ Studies in mice suggest additional NLRP3‐independent anti‐inflammatory mechanisms of actions for the compound, but in a phase 1 clinical trial, OLT1177 showed a good pharmacokinetic profile in healthy individuals with long half‐life and no hematological or organ toxicity at any of the tested doses.^130^ A recently completed phase 2 proof of concept study suggests that OLT1177 may have significant anti‐inflammatory activity in patients suffering from acute gout.^131^

CY‐09 is an analog of a cystic fibrosis transmembrane conductance regulator (CFTR) channel inhibitor that was found to inhibit the NLRP3 inflammasome with μM IC_50_ values without affecting NLRP3 priming.^132^ Unlike the parental molecule, CY‐09 failed to inhibit CFTR activity and was shown to specifically target the ATPase activity of NLRP3 by binding at the walker A motif in the central NACHT domain (Figure 3). Concordantly, CY‐09 significantly decreased serum levels of IL‐1β and neutrophil influx in a murine model of peritoneal MSU‐induced inflammation, and it protected mice from neonatal lethality upon in vivo expression of an MWS‐associated NLRP3 mutant.^132^

3,4‐methylenedioxy‐β‐nitrostyrene (MNS), a known inhibitor of tyrosine kinases, emerged as a hit for NLRP3 from a focused kinase inhibitor library screen.^133^ It interferes with NLRP3's ATPase activity by binding to the central NACHT and carboxy‐terminal LRR domains of NLRP3 (Figure 3). The acrylamide derivative INF58 irreversibly inhibits NLRP3 by covalently modifying Cys residues in its target protein. It was shown to inhibit ATPase activity of human recombinant NLRP3 and IL‐1β release from primary and immortalized macrophages.^134^

Unlike the previously mentioned synthetic molecules, oridonin is a natural compound derived from the medicinal plant *Rabdosia rubescens*. It was shown to inhibit NLRP3 activation by preventing the interaction between NLRP3 and NEK7 through covalent binding to Cys279 in the NACHT domain of NLRP3 (Figure 3). Furthermore, oridonin inhibited IL‐1β production in NLRP3‐dependent mouse models of peritonitis and gouty arthritis.^135^ However, in addition to interfering with NLRP3 inflammasome activation, the molecule was shown to inhibit inflammation through several additional mechanisms of action, including inhibition of NF‐κB and MAPK signaling, induction of apoptosis, cell cycle arrest and it was also shown to have anti‐tumor activity.^136^, ^137^, ^138^, ^139^

In addition to molecules with a direct mechanism of action on NLRP3, several molecules that inhibit NLRP3 activation indirectly by targeting post‐translational modifications have also been reported. Two recent studies suggest that acetylation of the amino‐terminal PYD in NLRP3 is required for recruitment of ASC and optimal downstream inflammasome signaling.^140^, ^141^ The authors go on to show that this mechanism was specific to NLRP3 because the AIM2 and NLRC4 inflammasomes were not affected by inhibition of this post‐translational modification. Furthermore, c‐Jun N‐terminal kinase1 (JNK1)‐mediated phosphorylation of NLRP3 Ser194 was shown to be a critical priming event that supports optimal NLRP3 activation.^34^ Although selective modulation of NLRP3 activation through these indirect mechanisms may prove challenging, potent pharmacological inhibitors that target specific post‐translational events might provide additional avenues to suppress NLRP3‐driven inflammation.

## 7 TARGETING ALTERNATIVE INFLAMMASOME PATHWAYS

In contrast to the breath of molecules that inhibit the NLRP3 inflammasome, selective small molecule inhibitors of other inflammasome sensors remain to be discovered. Nevertheless, the microtubule polymerization inhibitor colchicine selectively inhibits activation of the Pyrin inflammasome in murine macrophages and human monocytes.^78^ Interestingly, however, disease‐penetrant FMF mutations in *MEFV* render activation of the Pyrin inflammasome resistant to colchicine inhibition. This feature appeared highly specific for FMF‐associated mutations and was recently leveraged to develop a functional diagnostic assay for rapidly stratifying FMF patients.^80^ Significantly lower doses of colchicine are being used in the clinic since the 1970' to effectively control FMF symptoms, but the therapeutic mode of action of colchicine in FMF and gout patients remains a mystery.

Human NLRP1 and its murine paralogs Nlrp1a and Nlrp1b are unique in their requirement for proteasomal degradation to promote inflammasome activation.^26^, ^27^, ^142^, ^143^ In line herewith, proteasome inhibitors such as MG‐132 and bortezomib selectively inhibit activation of the NLRP1 inflammasome without modulating activation of NLRP3 and other inflammasome pathways.

## 8 SELECTIVE CASPASE‐1 INHIBITION—A REAL CHALLENGE

As the protease that matures IL‐1β and the common denominator of all canonical inflammasome pathways, developing clinical‐grade molecules that selectively inhibit caspase‐1 protease activity has been a focal effort of the pharmaceutical industry for many years. Like in other caspases, the evolutionary conserved Cys285 in the active site of caspase‐1 employs a nucleophilic attack. An electrophilic warhead that reacts with and inhibits Cys285 is therefore one of the strategies used by reversible (aldehyde‐containing) and non‐reversible (ketone‐based) tetrapeptide inhibitors to inhibit caspase‐1.^144^ Nonetheless, due to their toxic byproducts, poor stability, solubility and selectivity, tetrapeptide‐based inhibitors are not suitable for therapeutic application.^145^ Fully synthetic peptide‐mimetic prodrugs such VX‐740 (pralnacasan) and VX‐765 (belnacasan) were generated to overcome the toxicity and low bioavailability issues associated with peptidic caspase inhibitors. These prodrugs are metabolically converted in the cytosol by esterase activity to, respectively, VRT‐18858 and VRT‐043198, both of which act as reversible inhibitors of caspase‐1 and the related inflammatory caspases 4 and 5 (Figure 3).^146^, ^147^

VX‐740 was shown to reduce joint damage in two mouse models of osteoarthritis and it attenuated dextran sulfate sodium (DSS)‐induced colitis.^147^, ^148^ However, a phase 2 clinical trial with VX‐740 in rheumatoid arthritis patients was discontinued after hepatotoxicity was observed in long‐term follow‐up animal studies.^145^ VX‐765, on the other hand, was shown to reduce acute seizures and chronic epileptic activity in two mouse models of epilepsy, and it significantly suppressed the production of inflammatory mediators in models of rheumatoid arthritis and skin inflammation.^149^ It was also shown to improve cognition in a mouse model of Alzheimer's disease by inhibiting amyloid beta deposition and neuroinflammation.^150^ Although VX765 has a favorable safety profile, a phase 2 clinical trial in patients with treatment‐resistant partial epilepsy failed to reach its primary endpoints (ClinicalTrials.gov Identifier: NCT01501383).^151^ These challenges have spurred investigators to refocus efforts on inhibiting inflammasome sensors that act upstream of caspase‐1, and more recently, on targeting the pyroptosis effector GSDMD.

## 9 GSDMD—THE NEW KID ON THE BLOCK

GSDMD is expressed as a soluble, inactive precursor protein that consists of an amino‐terminal pore‐forming domain (GSDMD‐N) and a carboxy‐terminal auto‐inhibitory domain (GSDMD‐C) that are separated by a flexible interdomain linker.^9^, ^10^ Inflammatory caspases (ie, caspases 1, 4, and 5 in humans; and caspases 1 and 11 in mice) cleave GSDMD in the interdomain linker, thus allowing GSDMD‐N to translocate into the inner plasma membrane leaflet. It subsequently assembles into oligomers that pinch both plasma membrane leaflets to form large GSDMD pores allowing osmotic swelling and pyroptotic cell death.^152^ In addition to pyroptosis, GSDMD was shown to induce the release of web‐like chromatin structures called “neutrophil extracellular traps” from neutrophils as part of a specialized cell death mode known as NETosis.^153^, ^154^

Mice deficient in GSDMD are significantly protected against LPS‐induced endotoxic shock and lethality.^9^ Moreover, GSDMD was shown to play a critical role in driving IL‐1β‐dependent inflammatory pathology in mouse models of CAPS and FMF.^17^, ^18^ Moreover, preventing GSDMD activation in peripheral myeloid cells was recently shown to protect mice from neuroinflammation and demyelination in the EAE model of multiple sclerosis.^113^ Another recent study found a key role for GSDMD in non‐alcoholic steatohepatitis in humans and mice.^155^

These findings raised significant interest in the discovery of GSDMD‐targeted drugs, and several molecules have already been reported to inhibit GSDMD‐mediated pore formation. The alkylating compound necrosulfonamide (NSA) binds directly to full‐length GSDMD via Cys191, thereby preventing GSDMD‐N dimers from assembling higher order oligomers that drive pyroptotic pore formation and cell lysis (Figure 3). NSA similarly modifies a reactive Cys residue in mixed lineage kinase domain‐like pseudokinase (MLKL), thus preventing its recruitment to RIPK3 and inhibiting induction of another lytic cell death mode termed necroptosis.^156^, ^157^

Disulfiram and Bay 11‐7082 were recently discovered in a screen for inhibitors of GSDMD using a fluorogenic liposome leakage assay.^127^ Notably, both molecules inhibited GSDMD by covalently modifying the GSDMD Cys191 residue that is also targeted by NSA (Figure 3).^127^ As discussed above, Bay 11‐7082 is a potent inhibitor of inhibitor κB kinase (IKK) that also targets NLRP3 and caspase‐1.^126^ As an inhibitor of acetaldehyde dehydrogenase, disulfiram is used in the clinic for treating alcohol addiction.^158^ Disulfiram also inhibited TLR‐induced priming at higher concentrations, and both Disulfiram and Bay 11‐7082 attenuated ASC speck formation and the protease activities of caspases 1 and 11.^126^, ^127^, ^159^ Disulfiram was shown to inhibit pyroptosis in human and murine myeloid cells with µM potency, and it prevented secretion of IL‐1β from these cells.^127^ When dosed intraperitoneally in LPS‐challenged mice, it significantly delayed lethality at a concentration of 50 mg/kg.^127^

LDC7559 emerged as a direct inhibitor of GSDMD from a small molecule screen for inhibitors of phorbol ester‐induced NETosis in human neutrophils (Figure 3).^153^. Whereas disulfiram, NSA and Bay 11‐7082 inhibit GSDMD through covalent modification of Cys191, the mechanism of action of LDC7559 is unclear. LDC7559 inhibited pyroptosis by the NLRP3 and AIM2 inflammasomes,^153^ but other findings suggest that it may lack activity on recombinant GSDMD in the liposome leakage assay.^160^ Future studies should shed light on this apparent paradox.

Altogether, these findings highlight GSDMD as an attractive novel target to modulate pathological inflammation driven by pyroptosis, including the release of inflammasome‐dependent cytokines and DAMPs. While the current GSDMD tool compounds may still be a long way from the clinic, they may set the stage for the development of selective GSDMD‐targeted therapies by helping to shed light on the mechanisms that regulate GSDMD pore formation.

## 10 ACTIVATING INFLAMMASOME PATHWAYS IN CANCER THERAPY

As discussed above, results from CANTOS suggest that canakinumab‐mediated blockage of IL‐1β activity protects against the development of lung cancer, although the mechanism involved remains unclear. However, IL‐1β may have opposing roles in established tumors.^91^ Evidence that IL‐1β may have anti‐tumor activity was first presented 30 years ago, when intraperitoneal injection of recombinant human IL‐1β induced T‐cell‐dependent regression of subcutaneous SA1 sarcoma and L5178Y lymphomas in mice.^161^ The cancer‐suppressive effect of IL‐1β was confirmed in a mouse model for myeloma showing that IL‐1β (as well as IL‐1α) neutralization by anakinra severely impaired myeloma clearance by tumor‐specific Th1 cells and tumor‐infiltrating macrophages.^162^ Additionally, IL‐1β release from dendritic cells was shown to be essential for priming of interferon gamma (IFNγ)‐producing CD8^+^ T cells and elimination of tumor cells.^163^

Researchers are also investigating whether inflammasome activation and the induction of pyroptosis upstream of IL‐1β release can help render tumors more sensitive to therapy. Inhibitors of immune checkpoint modulators such as programmed death 1 (PD‐1) and cytotoxic T‐lymphocyte antigen 4 (CTLA‐4) invigorate CD8^+^ T cell‐mediated killing of tumor cells and are revolutionizing immunotherapy of cancer. However, only a minority of patients responds to checkpoint blockade due to a lack of CD8^+^ T‐cells in the tumor microenvironment. Researchers are exploring combination therapies that facilitate CD8^+^ T‐cell intratumoral infiltration in order to sensitize tumors to checkpoint therapy. In line with this strategy, an investigational phase I clinical trial in patients with solid cancers has recently been initiated with the NLRP3 agonist BMS‐986299 to study its therapeutic potential as a pyroptosis‐stimulating compound alone or when dosed to patients in combination with the checkpoint inhibitors Nivolumab and Ipilimumab (ClinicalTrials.gov Identifier: NCT03444753) (Figure 3).

Another clinical study aims to clarify the therapeutic effect of pyroptosis induction through the NLRP1 inflammasome in patients diagnosed with acute myeloid leukemia (AML), a heterogenous hematological malignancy driven by uncontrolled expansion of myeloid progenitor cells in bone marrow and in circulation. This study centers on the experimental broad‐spectrum DPP inhibitor BXCL701 (also named VbP, PT‐100, or Talabostat) that was recently assigned orphan drug designation for the treatment of AML by the FDA, and is based on recent findings showing that VbP/BXCL701 and more selective DPP8/DPP9 inhibitors are potent inducers of pyroptosis in AML cell lines and in primary AML samples from patients.^164^

Several recent studies showed that enforced activation of Gasdermin family members in cancer cells enhanced immune cell‐mediated tumor clearance in mouse models,^165^, ^166^, ^167^ adding credence to the notion that pyroptosis induction may enhance cancer immunotherapies to the benefit of patients.

## 11 CONCLUDING REMARKS

It has now become evident that inflammasomes play a crucial role in the immune response during microbial infections and non‐infectious diseases. A wealth of information regarding the complexity of inflammasome signaling and their roles in diseases has emerged during the past two decades. This has spurred interest in translating basic research findings into potential novel therapies for inflammasome‐driven autoinflammatory diseases. Moreover, intriguing findings from the CANTOS trial suggest that inflammasomes may contribute importantly to cardiovascular risk in atherosclerosis patients and to the development of lung cancer. Without doubt, the ongoing development of clinical inflammasome agonists and inhibitors will help tremendously to gain further insights into inflammasome‐driven autoinflammatory pathologies in the future and may expand treatment options for patients suffering from a host of common diseases with high unmet need, including metabolic and neurodegenerative diseases as well as certain cancers.

## CONFLICT OF INTEREST

D. Chauhan is an employee of Janssen Pharmaceutica N.V. The authors declare no conflict of interest.

## References

[1] Newton K , Dixit VM . Signaling in innate immunity and inflammation. Cold Spring Harb Perspect Biol. 2012;4(3):a006049. 22296764 10.1101/cshperspect.a006049 PMC3282411

[2] Meissner F , Scheltema RA , Mollenkopf HJ , Mann M . Direct proteomic quantification of the secretome of activated immune cells. Science. 2013;340:475‐478. 23620052 10.1126/science.1232578

[3] Broz P , Dixit VM . Inflammasomes: mechanism of assembly, regulation and signalling. Nat Rev Immunol. 2016;16:407‐420. 27291964 10.1038/nri.2016.58

[4] Lamkanfi M , Dixit VM . Mechanisms and functions of inflammasomes. Cell. 2014;157:1013‐1022. 24855941 10.1016/j.cell.2014.04.007

[5] Lamkanfi M , Declercq W , Kalai M , Saelens X , Vandenabeele P . Alice in caspase land. A phylogenetic analysis of caspases from worm to man. Cell Death Differ. 2002;9:358‐361. 11965488 10.1038/sj.cdd.4400989

[6] Heymann MC , Winkler S , Luksch H , et al Human procaspase‐1 variants with decreased enzymatic activity are associated with febrile episodes and may contribute to inflammation via RIP2 and NF‐kappaB signaling. J Immunol. 2014;192:4379‐4385. 24706726 10.4049/jimmunol.1203524

[7] Reinke S , Linge M , Diebner HH , et al Non‐canonical caspase‐1 signaling drives RIP2‐dependent and TNF‐alpha‐mediated inflammation in vivo. Cell Rep. 2020;30:2501‐2511.e5. 32101731 10.1016/j.celrep.2020.01.090

[8] Van Opdenbosch N , Van Gorp H , Verdonckt M , et al. Caspase‐1 engagement and TLR‐induced c‐FLIP expression suppress ASC/caspase‐8‐dependent apoptosis by inflammasome sensors NLRP1b and NLRC4. Cell Rep. 2017;21:3427‐3444. 29262324 10.1016/j.celrep.2017.11.088 PMC5746600

[9] Kayagaki N , Stowe IB , Lee BL , et al. Caspase‐11 cleaves gasdermin D for non‐canonical inflammasome signalling. Nature. 2015;526:666‐671. 26375259 10.1038/nature15541

[10] Shi J , Zhao Y , Wang K , et al. Cleavage of GSDMD by inflammatory caspases determines pyroptotic cell death. Nature. 2015;526:660‐665. 26375003 10.1038/nature15514

[11] He WT , Wan H , Hu L , et al. Gasdermin D is an executor of pyroptosis and required for interleukin‐1beta secretion. Cell Res. 2015;25:1285‐1298. 26611636 10.1038/cr.2015.139 PMC4670995

[12] Aglietti RA , Estevez A , Gupta A , et al. GsdmD p30 elicited by caspase‐11 during pyroptosis forms pores in membranes. Proc Natl Acad Sci U S A. 2016;113:7858‐7863. 27339137 10.1073/pnas.1607769113 PMC4948338

[13] Ding J , Wang K , Liu W , et al. Pore‐forming activity and structural autoinhibition of the gasdermin family. Nature. 2016;535:111‐116. 27281216 10.1038/nature18590

[14] Liu X , Zhang Z , Ruan J , et al. Inflammasome‐activated gasdermin D causes pyroptosis by forming membrane pores. Nature. 2016;535:153‐158. 27383986 10.1038/nature18629 PMC5539988

[15] Lamkanfi M , Sarkar A , Vande Walle L , et al. Inflammasome‐dependent release of the alarmin HMGB1 in endotoxemia. J Immunol. 2010;185:4385‐4392. 20802146 10.4049/jimmunol.1000803 PMC3428148

[16] Gross O , Yazdi AS , Thomas CJ , et al. Inflammasome activators induce interleukin‐1alpha secretion via distinct pathways with differential requirement for the protease function of caspase‐1. Immunity. 2012;36:388‐400. 22444631 10.1016/j.immuni.2012.01.018

[17] Kanneganti A , Malireddi RKS , Saavedra PHV , et al. GSDMD is critical for autoinflammatory pathology in a mouse model of Familial Mediterranean Fever. J Exp Med. 2018;215:1519‐1529. 29793924 10.1084/jem.20172060 PMC5987922

[18] Xiao J , Wang C , Yao J‐C , et al. Gasdermin D mediates the pathogenesis of neonatal‐onset multisystem inflammatory disease in mice. PLOS Biol. 2018;16(11):e3000047. 30388107 10.1371/journal.pbio.3000047 PMC6235378

[19] Martinon F , Burns K , Tschopp J . The inflammasome: a molecular platform triggering activation of inflammatory caspases and processing of proIL‐beta. Mol Cell. 2002;10:417‐426. 12191486 10.1016/s1097-2765(02)00599-3

[20] Boyden ED , Dietrich WF . Nalp1b controls mouse macrophage susceptibility to anthrax lethal toxin. Nat Genet. 2006;38:240‐244. 16429160 10.1038/ng1724

[21] Masters SL , Gerlic M , Metcalf D , et al. NLRP1 inflammasome activation induces pyroptosis of hematopoietic progenitor cells. Immunity. 2012;37:1009‐1023. 23219391 10.1016/j.immuni.2012.08.027 PMC4275304

[22] Okondo MC , Johnson DC , Sridharan R , et al. DPP8 and DPP9 inhibition induces pro‐caspase‐1‐dependent monocyte and macrophage pyroptosis. Nat Chem Biol. 2017;13:46‐53. 27820798 10.1038/nchembio.2229 PMC5477230

[23] Gai K , Okondo MC , Rao SD , et al. DPP8/9 inhibitors are universal activators of functional NLRP1 alleles. Cell Death Dis. 2019;10:587. 31383852 10.1038/s41419-019-1817-5 PMC6683174

[24] Zhong FL , Robinson K , Teo DET , et al. Human DPP9 represses NLRP1 inflammasome and protects against autoinflammatory diseases via both peptidase activity and FIIND domain binding. J Biol Chem. 2018;293:18864‐18878. 30291141 10.1074/jbc.RA118.004350 PMC6295727

[25] de Vasconcelos NM , Vliegen G , Gonçalves A , et al. DPP8/DPP9 inhibition elicits canonical Nlrp1b inflammasome hallmarks in murine macrophages. Life Sci Alliance. 2019;2(1):e201900313. 30718379 10.26508/lsa.201900313 PMC6362307

[26] Chui AJ , Okondo MC , Rao SD , et al. N‐terminal degradation activates the NLRP1B inflammasome. Science. 2019;364:82‐85. 30872531 10.1126/science.aau1208 PMC6610862

[27] Sandstrom A , Mitchell PS , Goers L , Mu EW , Lesser CF , Vance RE . Functional degradation: a mechanism of NLRP1 inflammasome activation by diverse pathogen enzymes. Science. 2019;364:eaau1330. 30872533 10.1126/science.aau1330 PMC6532986

[28] Drutman SB , Haerynck F , Zhong FL , et al. Homozygous NLRP1 gain‐of‐function mutation in siblings with a syndromic form of recurrent respiratory papillomatosis. Proc Natl Acad Sci U S A. 2019;116:19055‐19063. 31484767 10.1073/pnas.1906184116 PMC6754618

[29] Zhong FL , Mamai O , Sborgi L , et al. Germline NLRP1 mutations cause skin inflammatory and cancer susceptibility syndromes via inflammasome activation. Cell. 2016;167:187‐202.e7. 27662089 10.1016/j.cell.2016.09.001

[30] Grandemange S , Sanchez E , Louis‐Plence S , et al. A new autoinflammatory and autoimmune syndrome associated with NLRP1 mutations: NAIAD (NLRP1‐associated autoinflammation with arthritis and dyskeratosis). Ann Rheum Dis. 2017;76:1191‐1198. 27965258 10.1136/annrheumdis-2016-210021

[31] Duncan JA , Bergstralh DT , Wang Y , et al. Cryopyrin/NALP3 binds ATP/dATP, is an ATPase, and requires ATP binding to mediate inflammatory signaling. Proc Natl Acad Sci U S A. 2007;104:8041‐8046. 17483456 10.1073/pnas.0611496104 PMC1876568

[32] Bauernfeind FG , Horvath G , Stutz A , et al Cutting edge: NF‐kappaB activating pattern recognition and cytokine receptors license NLRP3 inflammasome activation by regulating NLRP3 expression. J Immunol. 2009;183:787‐791. 19570822 10.4049/jimmunol.0901363 PMC2824855

[33] Py BF , Kim MS , Vakifahmetoglu‐Norberg H , Yuan J . Deubiquitination of NLRP3 by BRCC3 critically regulates inflammasome activity. Mol Cell. 2013;49:331‐338. 23246432 10.1016/j.molcel.2012.11.009

[34] Song N , Liu Z‐S , Xue W , et al. NLRP3 phosphorylation is an essential priming event for inflammasome activation. Mol Cell. 2017;68:185‐197.e6. 28943315 10.1016/j.molcel.2017.08.017

[35] Lin KM , Hu W , Troutman TD , et al. IRAK‐1 bypasses priming and directly links TLRs to rapid NLRP3 inflammasome activation. Proc Natl Acad Sci U S A. 2014;111:775‐780. 24379360 10.1073/pnas.1320294111 PMC3896167

[36] Juliana C , Fernandes‐Alnemri T , Kang S , Farias A , Qin F , Alnemri ES . Non‐transcriptional priming and deubiquitination regulate NLRP3 inflammasome activation. J Biol Chem. 2012;287:36617‐36622. 22948162 10.1074/jbc.M112.407130 PMC3476327

[37] Fernandes‐Alnemri T , Kang S , Anderson C , Sagara J , Fitzgerald KA , Alnemri ES . Cutting edge: TLR signaling licenses IRAK1 for rapid activation of the NLRP3 inflammasome. J Immunol. 2013;191:3995‐3999. 24043892 10.4049/jimmunol.1301681 PMC3924784

[38] Mayor A , Martinon F , De Smedt T , Petrilli V , Tschopp J . A crucial function of SGT1 and HSP90 in inflammasome activity links mammalian and plant innate immune responses. Nat Immunol. 2007;8:497‐503. 17435760 10.1038/ni1459

[39] He Y , Zeng MY , Yang D , Motro B , Nunez G . NEK7 is an essential mediator of NLRP3 activation downstream of potassium efflux. Nature. 2016;530:354‐357. 26814970 10.1038/nature16959 PMC4810788

[40] Shi H , Wang Y , Li X , et al. NLRP3 activation and mitosis are mutually exclusive events coordinated by NEK7, a new inflammasome component. Nat Immunol. 2016;17:250‐258. 26642356 10.1038/ni.3333 PMC4862588

[41] Schmid‐Burgk JL , Chauhan D , Schmidt T , et al. A genome‐wide CRISPR (clustered regularly interspaced short palindromic repeats) screen identifies NEK7 as an essential component of NLRP3 inflammasome activation. J Biol Chem. 2016;291:103‐109. 26553871 10.1074/jbc.C115.700492 PMC4697147

[42] Schmacke NA , Gaidt MM , Szymanska I , et alPriming enables a NEK7‐independent route of NLRP3 activation. bioRxiv. 2019 10.1101/799320

[43] Munoz‐Planillo R , Kuffa P , Martinez‐Colon G , Smith BL , Rajendiran TM , Nunez G . K(+) efflux is the common trigger of NLRP3 inflammasome activation by bacterial toxins and particulate matter. Immunity. 2013;38:1142‐1153. 23809161 10.1016/j.immuni.2013.05.016 PMC3730833

[44] Di A , Xiong S , Ye Z , et al. The TWIK2 potassium efflux channel in macrophages mediates NLRP3 inflammasome‐induced inflammation. Immunity. 2018;49:56‐65.e4. 29958799 10.1016/j.immuni.2018.04.032 PMC6051907

[45] Petrilli V , Papin S , Dostert C , Mayor A , Martinon F , Tschopp J . Activation of the NALP3 inflammasome is triggered by low intracellular potassium concentration. Cell Death Differ. 2007;14:1583‐1589. 17599094 10.1038/sj.cdd.4402195

[46] Martinon F , Petrilli V , Mayor A , Tardivel A , Tschopp J . Gout‐associated uric acid crystals activate the NALP3 inflammasome. Nature. 2006;440:237‐241. 16407889 10.1038/nature04516

[47] Duewell P , Kono H , Rayner KJ , et al. NLRP3 inflammasomes are required for atherogenesis and activated by cholesterol crystals. Nature. 2010;464:1357‐1361. 20428172 10.1038/nature08938 PMC2946640

[48] Vande Walle L , Van Opdenbosch N , Jacques P , et al. Negative regulation of the NLRP3 inflammasome by A20 protects against arthritis. Nature. 2014;512:69‐73. 25043000 10.1038/nature13322 PMC4126806

[49] Wree A , McGeough MD , Peña CA , et al. NLRP3 inflammasome activation is required for fibrosis development in NAFLD. J Mol Med (Berl). 2014;92:1069‐1082. 24861026 10.1007/s00109-014-1170-1 PMC4349416

[50] Vandanmagsar B , Youm Y‐H , Ravussin A , et al. The NLRP3 inflammasome instigates obesity‐induced inflammation and insulin resistance. Nat Med. 2011;17:179‐188. 21217695 10.1038/nm.2279 PMC3076025

[51] Heneka MT , Kummer MP , Stutz A , et al. NLRP3 is activated in Alzheimer's disease and contributes to pathology in APP/PS1 mice. Nature. 2013;493:674‐678. 23254930 10.1038/nature11729 PMC3812809

[52] Gordon R , Albornoz EA , Christie DC , et al. Inflammasome inhibition prevents alpha‐synuclein pathology and dopaminergic neurodegeneration in mice. Sci Transl Med. 2018;10:456. 10.1126/scitranslmed.aah4066 PMC6483075 30381407

[53] Gris D , Ye Z , Iocca HA , et al. NLRP3 plays a critical role in the development of experimental autoimmune encephalomyelitis by mediating Th1 and Th17 responses. J Immunol. 2010;185:974‐981. 20574004 10.4049/jimmunol.0904145 PMC3593010

[54] Jha S , Srivastava SY , Brickey WJ , et al. The inflammasome sensor, NLRP3, regulates CNS inflammation and demyelination via caspase‐1 and interleukin‐18. J Neurosci. 2010;30:15811‐15820. 21106820 10.1523/JNEUROSCI.4088-10.2010 PMC6633756

[55] Zheng M , Karki R , Vogel P , Kanneganti TD . Caspase‐6 is a key regulator of innate immunity, inflammasome activation, and host defense. Cell. 2020;181:674‐687.e13. 32298652 10.1016/j.cell.2020.03.040 PMC7425208

[56] Malireddi RKS , Gurung P , Kesavardhana S , et al. Innate immune priming in the absence of TAK1 drives RIPK1 kinase activity‐independent pyroptosis, apoptosis, necroptosis, and inflammatory disease. J Exp Med. 2020;217(3):e20191644. 31869420 10.1084/jem.20191644 PMC7062518

[57] Malireddi RKS , Gurung P , Mavuluri J , et al. TAK1 restricts spontaneous NLRP3 activation and cell death to control myeloid proliferation. J Exp Med. 2018;215:1023‐1034. 29500178 10.1084/jem.20171922 PMC5881469

[58] Christgen S , Zheng M , Kesavardhana S , et al. Identification of the PANoptosome: a molecular platform triggering pyroptosis, apoptosis, and necroptosis (PANoptosis). Front Cell Infect Microbiol. 2020;10:237. 32547960 10.3389/fcimb.2020.00237 PMC7274033

[59] Samir P , Malireddi RKS , Kanneganti TD . The PANoptosome: a deadly protein complex driving pyroptosis, apoptosis, and necroptosis (PANoptosis). Front Cell Infect Microbiol. 2020;10:238. 32582562 10.3389/fcimb.2020.00238 PMC7283380

[60] Van Gorp H , Van Opdenbosch N , Lamkanfi M . Inflammasome‐dependent cytokines at the crossroads of health and autoinflammatory disease. Cold Spring Harb Perspect Biol. 2019;11(1):a028563. 29038114 10.1101/cshperspect.a028563 PMC6314066

[61] Brydges SD , Broderick L , McGeough MD , Pena CA , Mueller JL , Hoffman HM . Divergence of IL‐1, IL‐18, and cell death in NLRP3 inflammasomopathies. J Clin Invest. 2013;123:4695‐4705. 24084736 10.1172/JCI71543 PMC3809806

[62] Zhao Y , Yang J , Shi J , et al. The NLRC4 inflammasome receptors for bacterial flagellin and type III secretion apparatus. Nature. 2011;477:596‐600. 21918512 10.1038/nature10510

[63] Yang J , Zhao Y , Shi J , Shao F . Human NAIP and mouse NAIP1 recognize bacterial type III secretion needle protein for inflammasome activation. Proc Natl Acad Sci U S A. 2013;110:14408‐14413. 23940371 10.1073/pnas.1306376110 PMC3761597

[64] Kofoed EM , Vance RE . Innate immune recognition of bacterial ligands by NAIPs determines inflammasome specificity. Nature. 2011;477:592‐595. 21874021 10.1038/nature10394 PMC3184209

[65] Canna SW , de Jesus AA , Gouni S , et al. An activating NLRC4 inflammasome mutation causes autoinflammation with recurrent macrophage activation syndrome. Nat Genet. 2014;46:1140‐1146. 25217959 10.1038/ng.3089 PMC4177369

[66] Romberg N , Al Moussawi K , Nelson‐Williams C , et al. Mutation of NLRC4 causes a syndrome of enterocolitis and autoinflammation. Nat Genet. 2014;46:1135‐1139. 25217960 10.1038/ng.3066 PMC4177367

[67] Hornung V , Ablasser A , Charrel‐Dennis M , et al. AIM2 recognizes cytosolic dsDNA and forms a caspase‐1‐activating inflammasome with ASC. Nature. 2009;458:514‐518. 19158675 10.1038/nature07725 PMC2726264

[68] Fernandes‐Alnemri T , Yu J‐W , Juliana C , et al. The AIM2 inflammasome is critical for innate immunity to *Francisella tularensis* . Nat Immunol. 2010;11:385‐393. 20351693 10.1038/ni.1859 PMC3111085

[69] Sauer JD , Witte CE , Zemansky J , Hanson B , Lauer P , Portnoy DA . Listeria monocytogenes triggers AIM2‐mediated pyroptosis upon infrequent bacteriolysis in the macrophage cytosol. Cell Host Microbe. 2010;7:412‐419. 20417169 10.1016/j.chom.2010.04.004 PMC2947455

[70] Jones JW , Kayagaki N , Broz P , et al. Absent in melanoma 2 is required for innate immune recognition of *Francisella tularensis* . Proc Natl Acad Sci U S A. 2010;107:9771‐9776. 20457908 10.1073/pnas.1003738107 PMC2906881

[71] Burckstummer T , Baumann C , Blüml S , et al. An orthogonal proteomic‐genomic screen identifies AIM2 as a cytoplasmic DNA sensor for the inflammasome. Nat Immunol. 2009;10:266‐272. 19158679 10.1038/ni.1702

[72] Man SM , Karki R , Malireddi RKS , et al. The transcription factor IRF1 and guanylate‐binding proteins target activation of the AIM2 inflammasome by Francisella infection. Nat Immunol. 2015;16:467‐475. 25774715 10.1038/ni.3118 PMC4406811

[73] Meunier E , Wallet P , Dreier RF , et al. Guanylate‐binding proteins promote activation of the AIM2 inflammasome during infection with *Francisella novicida* . Nat Immunol. 2015;16:476‐484. 25774716 10.1038/ni.3119 PMC4568307

[74] Hu B , Jin C , Li H‐B , et al. The DNA‐sensing AIM2 inflammasome controls radiation‐induced cell death and tissue injury. Science. 2016;354:765‐768. 27846608 10.1126/science.aaf7532 PMC5640175

[75] Gaidt MM , Ebert TS , Chauhan D , et al. The DNA inflammasome in human myeloid cells is initiated by a STING‐cell death program upstream of NLRP3. Cell. 2017;171:1110‐1124.e18. 29033128 10.1016/j.cell.2017.09.039 PMC5901709

[76] Xu H , Yang J , Gao W , et al. Innate immune sensing of bacterial modifications of Rho GTPases by the Pyrin inflammasome. Nature. 2014;513:237‐241. 24919149 10.1038/nature13449

[77] Gao W , Yang J , Liu W , Wang Y , Shao F . Site‐specific phosphorylation and microtubule dynamics control Pyrin inflammasome activation. Proc Natl Acad Sci U S A. 2016;113:E4857‐E4866. 27482109 10.1073/pnas.1601700113 PMC4995971

[78] Van Gorp H , Saavedra PHV , de Vasconcelos NM , et al. Familial Mediterranean fever mutations lift the obligatory requirement for microtubules in Pyrin inflammasome activation. Proc Natl Acad Sci U S A. 2016;113:14384‐14389. 27911804 10.1073/pnas.1613156113 PMC5167202

[79] Masters SL , Lagou V , Jéru I , et al. Familial autoinflammation with neutrophilic dermatosis reveals a regulatory mechanism of pyrin activation. Sci Transl Med. 2016;8:332ra345. 10.1126/scitranslmed.aaf1471 27030597

[80] Van Gorp H , Huang L , Saavedra P , et al. Blood‐based test for diagnosis and functional subtyping of familial Mediterranean fever. Ann Rheum Dis. 2020;79:960‐968. 32312770 10.1136/annrheumdis-2019-216701 PMC7307214

[81] Kayagaki N , Warming S , Lamkanfi M , et al. Non‐canonical inflammasome activation targets caspase‐11. Nature. 2011;479:117‐121. 22002608 10.1038/nature10558

[82] Hagar JA , Powell DA , Aachoui Y , Ernst RK , Miao EA . Cytoplasmic LPS activates caspase‐11: implications in TLR4‐independent endotoxic shock. Science. 2013;341:1250‐1253. 24031018 10.1126/science.1240988 PMC3931427

[83] Kayagaki N , Wong MT , Stowe IB , et al. Noncanonical inflammasome activation by intracellular LPS independent of TLR4. Science. 2013;341:1246‐1249. 23887873 10.1126/science.1240248

[84] Shi J , Zhao Y , Wang Y , et al. Inflammatory caspases are innate immune receptors for intracellular LPS. Nature. 2014;514:187‐192. 25119034 10.1038/nature13683

[85] Baker PJ , Boucher D , Bierschenk D , et al. NLRP3 inflammasome activation downstream of cytoplasmic LPS recognition by both caspase‐4 and caspase‐5. Eur J Immunol. 2015;45:2918‐2926. 26173988 10.1002/eji.201545655

[86] Schmid‐Burgk JL , Gaidt MM , Schmidt T , Ebert TS , Bartok E , Hornung V . Caspase‐4 mediates non‐canonical activation of the NLRP3 inflammasome in human myeloid cells. Eur J Immunol. 2015;45:2911‐2917. 26174085 10.1002/eji.201545523

[87] Knodler LA , Crowley SM , Sham HP , et al. Noncanonical inflammasome activation of caspase‐4/caspase‐11 mediates epithelial defenses against enteric bacterial pathogens. Cell Host Microbe. 2014;16:249‐256. 25121752 10.1016/j.chom.2014.07.002 PMC4157630

[88] Lagrange B , Benaoudia S , Wallet P , et al. Human caspase‐4 detects tetra‐acylated LPS and cytosolic Francisella and functions differently from murine caspase‐11. Nat Commun. 2018;9:242. 29339744 10.1038/s41467-017-02682-y PMC5770465

[89] Mandal P , Feng Y , Lyons JD , et al. Caspase‐8 collaborates with caspase‐11 to drive tissue damage and execution of endotoxic shock. Immunity. 2018;49:42‐55.e6. 30021146 10.1016/j.immuni.2018.06.011 PMC6064639

[90] Dinarello CA . A clinical perspective of IL‐1beta as the gatekeeper of inflammation. Eur J Immunol. 2011;41:1203‐1217. 21523780 10.1002/eji.201141550

[91] Van Gorp H , Lamkanfi M . The emerging roles of inflammasome‐dependent cytokines in cancer development. EMBO Rep. 2019;20:e47575. 31101676 10.15252/embr.201847575 PMC6549028

[92] Voet S , Srinivasan S , Lamkanfi M , van Loo G . Inflammasomes in neuroinflammatory and neurodegenerative diseases. EMBO Mol Med. 2019;11:e10248. 31015277 10.15252/emmm.201810248 PMC6554670

[93] Lamkanfi M . Emerging inflammasome effector mechanisms. Nat Rev Immunol. 2011;11:213‐220. 21350580 10.1038/nri2936

[94] Dinarello CA . Immunological and inflammatory functions of the interleukin‐1 family. Annu Rev Immunol. 2009;27:519‐550. 19302047 10.1146/annurev.immunol.021908.132612

[95] Colotta F , Re F , Muzio M , et al. Interleukin‐1 type II receptor: a decoy target for IL‐1 that is regulated by IL‐4. Science. 1993;261:472‐475. 8332913 10.1126/science.8332913

[96] Bode JG , Albrecht U , Haussinger D , Heinrich PC , Schaper F . Hepatic acute phase proteins–regulation by IL‐6‐ and IL‐1‐type cytokines involving STAT3 and its crosstalk with NF‐kappaB‐dependent signaling. Eur J Cell Biol. 2012;91:496‐505. 22093287 10.1016/j.ejcb.2011.09.008

[97] Ben‐Sasson SZ , Wang K , Cohen J , Paul WE . IL‐1beta strikingly enhances antigen‐driven CD4 and CD8 T‐cell responses. Cold Spring Harb Symp Quant Biol. 2013;78:117‐124. 24092469 10.1101/sqb.2013.78.021246

[98] Santarlasci V , Cosmi L , Maggi L , Liotta F , Annunziato F . IL‐1 and T helper immune responses. Front Immunol. 2013;4:182. 23874332 10.3389/fimmu.2013.00182 PMC3711056

[99] Janssen CA , Oude Voshaar MAH , Vonkeman HE , et al. Anakinra for the treatment of acute gout flares: a randomized, double‐blind, placebo‐controlled, active‐comparator, non‐inferiority trial. Rheumatology. 2019;58(8):1344‐1352. 10.1093/rheumatology/key402 30602035

[100] Ruscitti P , Masedu F , Alvaro S , et al. Anti‐interleukin‐1 treatment in patients with rheumatoid arthritis and type 2 diabetes (TRACK): a multicentre, open‐label, randomised controlled trial. PLoS Med. 2019;16:e1002901. 31513665 10.1371/journal.pmed.1002901 PMC6742232

[101] De Benedetti F , Gattorno M , Anton J , et al. Canakinumab for the treatment of autoinflammatory recurrent fever syndromes. N Engl J Med. 2018;378:1908‐1919. 29768139 10.1056/NEJMoa1706314

[102] Ridker PM , Everett BM , Thuren T , et al. Antiinflammatory therapy with canakinumab for atherosclerotic disease. N Engl J Med. 2017;377:1119‐1131. 28845751 10.1056/NEJMoa1707914

[103] Ridker PM , Howard CP , Walter V , et al. Effects of interleukin‐1beta inhibition with canakinumab on hemoglobin A1c, lipids, C‐reactive protein, interleukin‐6, and fibrinogen: a phase IIb randomized, placebo‐controlled trial. Circulation. 2012;126:2739‐2748. 23129601 10.1161/CIRCULATIONAHA.112.122556

[104] Ridker PM , MacFadyen JG , Thuren T , et al. Effect of interleukin‐1beta inhibition with canakinumab on incident lung cancer in patients with atherosclerosis: exploratory results from a randomised, double‐blind, placebo‐controlled trial. Lancet. 2017;390:1833‐1842. 28855077 10.1016/S0140-6736(17)32247-X

[105] Hoffman HM , Throne ML , Amar NJ , et al. Long‐term efficacy and safety profile of rilonacept in the treatment of cryopryin‐associated periodic syndromes: results of a 72‐week open‐label extension study. Clin Ther. 2012;34:2091‐2103. 23031624 10.1016/j.clinthera.2012.09.009

[106] Krause K , Weller K , Stefaniak R , et al. Efficacy and safety of the interleukin‐1 antagonist rilonacept in Schnitzler syndrome: an open‐label study. Allergy. 2012;67:943‐950. 22583335 10.1111/j.1398-9995.2012.02843.x

[107] Kudela H , Drynda S , Lux A , Horneff G , Kekow J . Comparative study of Interleukin‐18 (IL‐18) serum levels in adult onset Still's disease (AOSD) and systemic onset juvenile idiopathic arthritis (sJIA) and its use as a biomarker for diagnosis and evaluation of disease activity. BMC Rheumatol. 2019;3:4. 10.1186/s41927-019-0053-z PMC6394042 30886992

[108] Weiss ES , Girard‐Guyonvarc'h C , Holzinger D , et al. Interleukin‐18 diagnostically distinguishes and pathogenically promotes human and murine macrophage activation syndrome. Blood. 2018;131:1442‐1455. 29326099 10.1182/blood-2017-12-820852 PMC5877443

[109] Gabay C , Fautrel B , Rech J , et al. Open‐label, multicentre, dose‐escalating phase II clinical trial on the safety and efficacy of tadekinig alfa (IL‐18BP) in adult‐onset Still's disease. Ann Rheum Dis. 2018;77:840‐847. 29472362 10.1136/annrheumdis-2017-212608 PMC5965361

[110] Canna SW , Girard C , Malle L , et al. Life‐threatening NLRC4‐associated hyperinflammation successfully treated with IL‐18 inhibition. J Allergy Clin Immunol. 2017;139:1698‐1701. 27876626 10.1016/j.jaci.2016.10.022 PMC5846100

[111] Ottaviani S , Moltó A , Ea H‐K , et al. Efficacy of anakinra in gouty arthritis: a retrospective study of 40 cases. Arthritis Res Ther. 2013;15:R123. 24432362 10.1186/ar4303 PMC3978950

[112] Brydges SD , Mueller JL , McGeough MD , et al. Inflammasome‐mediated disease animal models reveal roles for innate but not adaptive immunity. Immunity. 2009;30:875‐887. 19501000 10.1016/j.immuni.2009.05.005 PMC2759865

[113] Li S , Wu Y , Yang D , et al. Gasdermin D in peripheral myeloid cells drives neuroinflammation in experimental autoimmune encephalomyelitis. J Exp Med. 2019;216:2562‐2581. 31467036 10.1084/jem.20190377 PMC6829591

[114] Mangan MSJ , Olhava EJ , Roush WR , Seidel HM , Glick GD , Latz E . Targeting the NLRP3 inflammasome in inflammatory diseases. Nat Rev Drug Discov. 2018;17:588‐606. 30026524 10.1038/nrd.2018.97

[115] Lamkanfi M , Mueller JL , Vitari AC , et al. Glyburide inhibits the Cryopyrin/Nalp3 inflammasome. J Cell Biol. 2009;187:61‐70. 19805629 10.1083/jcb.200903124 PMC2762099

[116] Coll RC , Robertson AAB , Chae JJ , et al. A small‐molecule inhibitor of the NLRP3 inflammasome for the treatment of inflammatory diseases. Nat Med. 2015;21:248‐255. 25686105 10.1038/nm.3806 PMC4392179

[117] Tapia‐Abellan A , Angosto‐Bazarra D , Martínez‐Banaclocha H , et al. MCC950 closes the active conformation of NLRP3 to an inactive state. Nat Chem Biol. 2019;15:560‐564. 31086329 10.1038/s41589-019-0278-6 PMC7116292

[118] Coll RC , Hill JR , Day CJ , et al. MCC950 directly targets the NLRP3 ATP‐hydrolysis motif for inflammasome inhibition. Nat Chem Biol. 2019;15:556‐559. 31086327 10.1038/s41589-019-0277-7

[119] Vande Walle L , Stowe IB , Šácha P , et al. MCC950/CRID3 potently targets the NACHT domain of wild‐type NLRP3 but not disease‐associated mutants for inflammasome inhibition. PLoS Biol. 2019;17:e3000354. 31525186 10.1371/journal.pbio.3000354 PMC6762198

[120] van der Heijden T , Kritikou E , Venema W , et al. NLRP3 inflammasome inhibition by MCC950 reduces atherosclerotic lesion development in apolipoprotein E‐deficient mice‐brief report. Arterioscler Thromb Vasc Biol. 2017;37:1457‐1461. 28596375 10.1161/ATVBAHA.117.309575

[121] van Hout GP , Bosch L , Ellenbroek GHJM , et al. The selective NLRP3‐inflammasome inhibitor MCC950 reduces infarct size and preserves cardiac function in a pig model of myocardial infarction. Eur Heart J. 2017;38:828‐836. 27432019 10.1093/eurheartj/ehw247

[122] Primiano MJ , Lefker BA , Bowman MR , et al. Efficacy and pharmacology of the NLRP3 inflammasome inhibitor CP‐456,773 (CRID3) in murine models of dermal and pulmonary inflammation. J Immunol. 2016;197:2421‐2433. 27521339 10.4049/jimmunol.1600035

[123] Perera AP , Fernando R , Shinde T , et al. MCC950, a specific small molecule inhibitor of NLRP3 inflammasome attenuates colonic inflammation in spontaneous colitis mice. Sci Rep. 2018;8:8618. 29872077 10.1038/s41598-018-26775-w PMC5988655

[124] Dempsey C , Araiz AR , Bryson KJ , et al. Inhibiting the NLRP3 inflammasome with MCC950 promotes non‐phlogistic clearance of amyloid‐beta and cognitive function in APP/PS1 mice. Brain Behav Immun. 2017;61:306‐316. 28003153 10.1016/j.bbi.2016.12.014

[125] Bennett J , Basivireddy J , Kollar A , et al. Blood‐brain barrier disruption and enhanced vascular permeability in the multiple sclerosis model EAE. J Neuroimmunol. 2010;229:180‐191. 20832870 10.1016/j.jneuroim.2010.08.011

[126] Juliana C , Fernandes‐Alnemri T , Wu J , et al. Anti‐inflammatory compounds parthenolide and Bay 11–7082 are direct inhibitors of the inflammasome. J Biol Chem. 2010;285:9792‐9802. 20093358 10.1074/jbc.M109.082305 PMC2843228

[127] Hu JJ , Liu X , Zhao J , et alIdentification of pyroptosis inhibitors that target a reactive cysteine in gasdermin D. bioRxiv. 2018.

[128] Huang Y , Jiang H , Chen Y , et al. Tranilast directly targets NLRP3 to treat inflammasome‐driven diseases. EMBO Mol Med. 2018;10:e8689. 29531021 10.15252/emmm.201708689 PMC5887903

[129] Darakhshan S , Pour AB . Tranilast: a review of its therapeutic applications. Pharmacol Res. 2015;91:15‐28. 25447595 10.1016/j.phrs.2014.10.009

[130] Marchetti C , Swartzwelter B , Gamboni F , et al. OLT1177, a beta‐sulfonyl nitrile compound, safe in humans, inhibits the NLRP3 inflammasome and reverses the metabolic cost of inflammation. Proc Natl Acad Sci U S A. 2018;115:E1530‐E1539. 29378952 10.1073/pnas.1716095115 PMC5816172

[131] Jansen T , Kluck V , Janssen M , et al. The first phase 2a proof‐of‐concept study of a selective NLRP3 inflammasome inhibitor, dapansutrile (OLT1177), in acute gout [abstract]. Arthritis Rheumatol. 2019;71(Suppl 10).

[132] Jiang H , He H , Chen Y , et al. Identification of a selective and direct NLRP3 inhibitor to treat inflammatory disorders. J Exp Med. 2017;214:3219‐3238. 29021150 10.1084/jem.20171419 PMC5679172

[133] He Y , Varadarajan S , Munoz‐Planillo R , Burberry A , Nakamura Y , Nunez G . 3,4‐methylenedioxy‐beta‐nitrostyrene inhibits NLRP3 inflammasome activation by blocking assembly of the inflammasome. J Biol Chem. 2014;289:1142‐1150. 24265316 10.1074/jbc.M113.515080 PMC3887181

[134] Cocco M , Miglio G , Giorgis M , et al. Design, synthesis, and evaluation of acrylamide derivatives as direct NLRP3 inflammasome inhibitors. ChemMedChem. 2016;11:1790‐1803. 26990578 10.1002/cmdc.201600055

[135] He H , Jiang H , Chen Y , et al. Oridonin is a covalent NLRP3 inhibitor with strong anti‐inflammasome activity. Nat Commun. 2018;9:2550. 29959312 10.1038/s41467-018-04947-6 PMC6026158

[136] Zhao G , Zhang T , Ma X , et al. Oridonin attenuates the release of pro‐inflammatory cytokines in lipopolysaccharide‐induced RAW264.7 cells and acute lung injury. Oncotarget. 2017;8:68153‐68164. 28978105 10.18632/oncotarget.19249 PMC5620245

[137] Kuo LM , Kuo CY , Lin CY , Hung MF , Shen JJ , Hwang TL . Intracellular glutathione depletion by oridonin leads to apoptosis in hepatic stellate cells. Molecules. 2014;19:3327‐3344. 24647034 10.3390/molecules19033327 PMC6270846

[138] Xu Y , Xue Y , Wang Y , Feng D , Lin S , Xu L . Multiple‐modulation effects of Oridonin on the production of proinflammatory cytokines and neurotrophic factors in LPS‐activated microglia. Int Immunopharmacol. 2009;9:360‐365. 19185062 10.1016/j.intimp.2009.01.002

[139] Yang J , Jiang H , Wang C , et al. Oridonin triggers apoptosis in colorectal carcinoma cells and suppression of microRNA‐32 expression augments oridonin‐mediated apoptotic effects. Biomed Pharmacother. 2015;72:125‐134. 26054686 10.1016/j.biopha.2015.04.016

[140] He M , Chiang H‐H , Luo H , et al. An acetylation switch of the NLRP3 inflammasome regulates aging‐associated chronic inflammation and insulin resistance. Cell Metab. 2020;31(3):580‐591.e5. 32032542 10.1016/j.cmet.2020.01.009 PMC7104778

[141] Zhao K , Zhang Y , Xu X , et al. Acetylation is required for NLRP3 self‐aggregation and full activation of the inflammasome. bioRxiv. 2019.

[142] Squires RC , Muehlbauer SM , Brojatsch J . Proteasomes control caspase‐1 activation in anthrax lethal toxin‐mediated cell killing. J Biol Chem. 2007;282:34260‐34267. 17878154 10.1074/jbc.M705687200

[143] Wickliffe KE , Leppla SH , Moayeri M . Anthrax lethal toxin‐induced inflammasome formation and caspase‐1 activation are late events dependent on ion fluxes and the proteasome. Cell Microbiol. 2008;10:332‐343. 17850338 10.1111/j.1462-5822.2007.01044.x PMC2515708

[144] Thornberry NA , Rano TA , Peterson EP , et al. A combinatorial approach defines specificities of members of the caspase family and granzyme B. Functional relationships established for key mediators of apoptosis. J Biol Chem. 1997;272:17907‐17911. 9218414 10.1074/jbc.272.29.17907

[145] MacKenzie SH , Schipper JL , Clark AC . The potential for caspases in drug discovery. Curr Opin Drug Discov Devel. 2010;13:568‐576. PMC3289102 20812148

[146] Wannamaker W , Davies R , Namchuk M , et al. (S)‐1‐((S)‐2‐{[1‐(4‐amino‐3‐chloro‐phenyl)‐methanoyl]‐amino}‐3,3‐dimethyl‐butanoy l)‐pyrrolidine‐2‐carboxylic acid ((2R,3S)‐2‐ethoxy‐5‐oxo‐tetrahydro‐furan‐3‐yl)‐amide (VX‐765), an orally available selective interleukin (IL)‐converting enzyme/caspase‐1 inhibitor, exhibits potent anti‐inflammatory activities by inhibiting the release of IL‐1beta and IL‐18. J Pharmacol Exp Ther. 2007;321:509‐516. 17289835 10.1124/jpet.106.111344

[147] Rudolphi K , Gerwin N , Verzijl N , van der Kraan P , van den Berg W . Pralnacasan, an inhibitor of interleukin‐1beta converting enzyme, reduces joint damage in two murine models of osteoarthritis. Osteoarthritis Cartilage. 2003;11:738‐746. 13129693 10.1016/s1063-4584(03)00153-5

[148] Bauer C , Duewell P , Mayer C , et al. Colitis induced in mice with dextran sulfate sodium (DSS) is mediated by the NLRP3 inflammasome. Gut. 2010;59:1192‐1199. 20442201 10.1136/gut.2009.197822

[149] Maroso M , Balosso S , Ravizza T , et al. Interleukin‐1beta biosynthesis inhibition reduces acute seizures and drug resistant chronic epileptic activity in mice. Neurotherapeutics. 2011;8:304‐315. 21431948 10.1007/s13311-011-0039-z PMC3101825

[150] Flores J , Noel A , Foveau B , Lynham J , Lecrux C , LeBlanc AC . Caspase‐1 inhibition alleviates cognitive impairment and neuropathology in an Alzheimer's disease mouse model. Nat Commun. 2018;9:3916. 30254377 10.1038/s41467-018-06449-x PMC6156230

[151] Bialer M , Johannessen SI , Levy RH , Perucca E , Tomson T , White HS . Progress report on new antiepileptic drugs: a summary of the Eleventh Eilat Conference (EILAT XI). Epilepsy Res. 2013;103:2‐30. 23219031 10.1016/j.eplepsyres.2012.10.001

[152] de Vasconcelos NM , Lamkanfi M . Recent Insights on Inflammasomes, Gasdermin Pores, and Pyroptosis. Cold Spring Harb Perspect Biol. 2020;12(5):a036392. 31570336 10.1101/cshperspect.a036392 PMC7197430

[153] Sollberger G , Choidas A , Burn GL , et al. Gasdermin D plays a vital role in the generation of neutrophil extracellular traps. Sci Immunol. 2018;3(26):eaar6689. 30143555 10.1126/sciimmunol.aar6689

[154] Chen KW , Monteleone M , Boucher D , et al. Noncanonical inflammasome signaling elicits gasdermin D‐dependent neutrophil extracellular traps. Sci Immunol. 2018;3(26):eaar6676. 30143554 10.1126/sciimmunol.aar6676

[155] Xu B , Jiang M , Chu Y , et al. Gasdermin D plays a key role as a pyroptosis executor of non‐alcoholic steatohepatitis in humans and mice. J Hepatol. 2018;68:773‐782. 29273476 10.1016/j.jhep.2017.11.040

[156] Sun L , Wang H , Wang Z , et al. Mixed lineage kinase domain‐like protein mediates necrosis signaling downstream of RIP3 kinase. Cell. 2012;148:213‐227. 22265413 10.1016/j.cell.2011.11.031

[157] Liu S , Liu H , Johnston A , et al. MLKL forms disulfide bond‐dependent amyloid‐like polymers to induce necroptosis. Proc Natl Acad Sci U S A. 2017;114:E7450‐E7459. 28827318 10.1073/pnas.1707531114 PMC5594682

[158] Wright C , Moore RD . Disulfiram treatment of alcoholism. Am J Med. 1990;88:647‐655. 2189310 10.1016/0002-9343(90)90534-k

[159] Nobel CS , Kimland M , Nicholson DW , Orrenius S , Slater AF . Disulfiram is a potent inhibitor of proteases of the caspase family. Chem Res Toxicol. 1997;10:1319‐1324. 9437520 10.1021/tx970131m

[160] Hu JJ , Liu X , Xia S , et al. FDA‐approved disulfiram inhibits pyroptosis by blocking gasdermin D pore formation. Nat Immunol. 2020;21(7):736‐745. 32367036 10.1038/s41590-020-0669-6 PMC7316630

[161] North RJ , Neubauer RH , Huang JJ , Newton RC , Loveless SE . Interleukin 1‐induced, T cell‐mediated regression of immunogenic murine tumors. Requirement for an adequate level of already acquired host concomitant immunity. J Exp Med. 1988;168:2031‐2043. 3143799 10.1084/jem.168.6.2031 PMC2189148

[162] Haabeth OA , Lorvik KB , Yagita H , Bogen B , Corthay A . Interleukin‐1 is required for cancer eradication mediated by tumor‐specific Th1 cells. Oncoimmunology. 2016;5:e1039763. 26942052 10.1080/2162402X.2015.1039763 PMC4760324

[163] Ghiringhelli F , Apetoh L , Tesniere A , et al. Activation of the NLRP3 inflammasome in dendritic cells induces IL‐1beta‐dependent adaptive immunity against tumors. Nat Med. 2009;15:1170‐1178. 19767732 10.1038/nm.2028

[164] Johnson DC , Taabazuing CY , Okondo MC , et al. DPP8/DPP9 inhibitor‐induced pyroptosis for treatment of acute myeloid leukemia. Nat Med. 2018;24:1151‐1156. 29967349 10.1038/s41591-018-0082-y PMC6082709

[165] Zhou Z , He H , Wang K , et al. Granzyme A from cytotoxic lymphocytes cleaves GSDMB to trigger pyroptosis in target cells. Science. 2020;368:eaaz7548. 32299851 10.1126/science.aaz7548

[166] Wang Q , Wang Y , Ding J , et al. A bioorthogonal system reveals antitumour immune function of pyroptosis. Nature. 2020;579:421‐426. 32188939 10.1038/s41586-020-2079-1

[167] Zhang Z , Zhang Y , Xia S , et al. Gasdermin E suppresses tumour growth by activating anti‐tumour immunity. Nature. 2020;579:415‐420. 32188940 10.1038/s41586-020-2071-9 PMC7123794

